# Synthesis and structural studies of N-heterocyclic carbene Ag(I) and Hg(II) complexes and recognition of dihydrogen phosphate anion

**DOI:** 10.1038/s41598-017-07961-8

**Published:** 2017-08-08

**Authors:** Qingxiang Liu, Xiaoqiang Zhao, Zeliang Hu, Zhixiang Zhao, Hong Wang

**Affiliations:** Key Laboratory of Inorganic-Organic Hybrid Functional Materials Chemistry (Tianjin Normal University), Ministry of Education; Tianjin Key Laboratory of Structure and Performance for Functional Molecules; College of Chemistry, Tianjin Normal University, Tianjin, 300387 China

## Abstract

Bis-benzimidazolium salt (*S*)-2,2′-bis[2″-(*N*-Et-benzimidazoliumyl)ethoxy]-1,1′-binaphthyl hexafluorophosphate [(*S*)-L^1^H_2_]·(PF_6_)_2_ and bis-imidazolium salts (*S*)-2,2′-bis[2″-(*N*-R-imidazoliumyl)ethoxy]-1,1′-binaphthyl hexafluorophosphate [(*S*)-L^2^H_2_]·(PF_6_)_2_ and [(*S*)-L^3^H_2_]·(PF_6_)_2_ (R = ethyl or benzyl), as well as their five N-heterocyclic carbene Hg(II) and Ag(I) complexes such as [(*S*)-L^1^Hg(HgBr_4_)] (1), [(*S*)-L^2^Hg(HgBr_4_)] (2), [(*S*)-L^2^Hg(HgI_4_)] (3), {[(*S*)-L^2^Ag](PF_6_)}_n_ (4) and [(*S*)-L^3^Ag](PF_6_) (5) have been prepared and characterized. Each of complexes 1–3 consists of two rings (one 6-membered ring and one 11-membered ring), in which the oxygen atom in the ligand participates in coordination with Hg(II) ion. In complex 4, 1D helical polymeric chain is formed via biscarbene ligand (*S*)-L^2^ and Ag(I) ion. A 15-membered macrometallocycle is constructed through a ligand (*S*)-L^3^ and a Ag(I) ion in complex 5. Additionally, the selective recognition of H_2_PO_4_^−^ using complex 5 as a receptor was investigated on the basis of fluorescence and UV/vis spectroscopic titrations. The results indicate that complex 5 can distinguish effectively H_2_PO_4_^−^ from other anions.

## Introduction

Numerous previous work based on fluorescence method has been performed in order to develop receptors for anions^1–7^. In the field of anion recognition, dihydrogen phosphate plays a very important role due to its responsibility for the eutrophication of natural water sources^8^. Though many receptors of H_2_PO_4_^−^ have been reported^9, 10^, developing highly sensitive and selective receptors is still desired.

In the course of searching for suitable receptors, we are interested in cyclic N-heterocyclic carbene (NHC) metal complexes since they could be readily prepared and are stable toward heat, moisture and air^11–19^. In the reported receptors for anions, the main acting force between the receptor and the guest includes hydrogen bonds, anion-π, and coordinating interaction^20–31^. Whereas, the synergistic effect of cycle (namely, the result of the combined effects of several weak intermolecular interactions) might be the main acting force upon using cyclic complexes as receptors. As described in some reported relative examples^32–34^, the cyclic NHC metal complexes show great potential application in the host-guest chemistry. We herein report the synthesis of bis-azolium salts (*S*)-2,2′-bis[2″-(*N*-R-azoliumyl)ethoxy]-1,1′-binaphthyl hexafluorophosphate [(***S***)**-L** ^**1**^ **H**_**2**_]**·**(**PF**_**6**_)_**2**_ ~ [(***S***)**-L** ^**3**^ **H**_**2**_]**·**(**PF**_**6**_)_**2**_ (R = ethyl or benzyl, azoliumyl = benzimidazoliumyl or imidazoliumyl) and the preparation and structure of five NHC mercury(II) and silver(I) complexes, [(*S*)-L^1^Hg(HgBr_4_)] (**1**), [(*S*)-L^2^Hg(HgBr_4_)] (**2**), [(*S*)-L^2^Hg(HgI_4_)] (**3**), {[(*S*)-L^2^Ag](PF_6_)}_n_ (**4**) and [(*S*)-L^3^Ag](PF_6_) (**5**). In addition, selective recognition of dihydrogen phosphate using cyclic NHC-Ag(I) complex **5** as a receptor is investigated on the basis of fluorescence and UV/vis spectroscopic titrations.

## Results and Discussion

### Synthesis and characterizations of precursors [(*S*)-L^1^H_2_]·(PF_6_)_2_ ~ [(*S*)-L^3^H_2_]·(PF_6_)_2_

**Figure 1:**
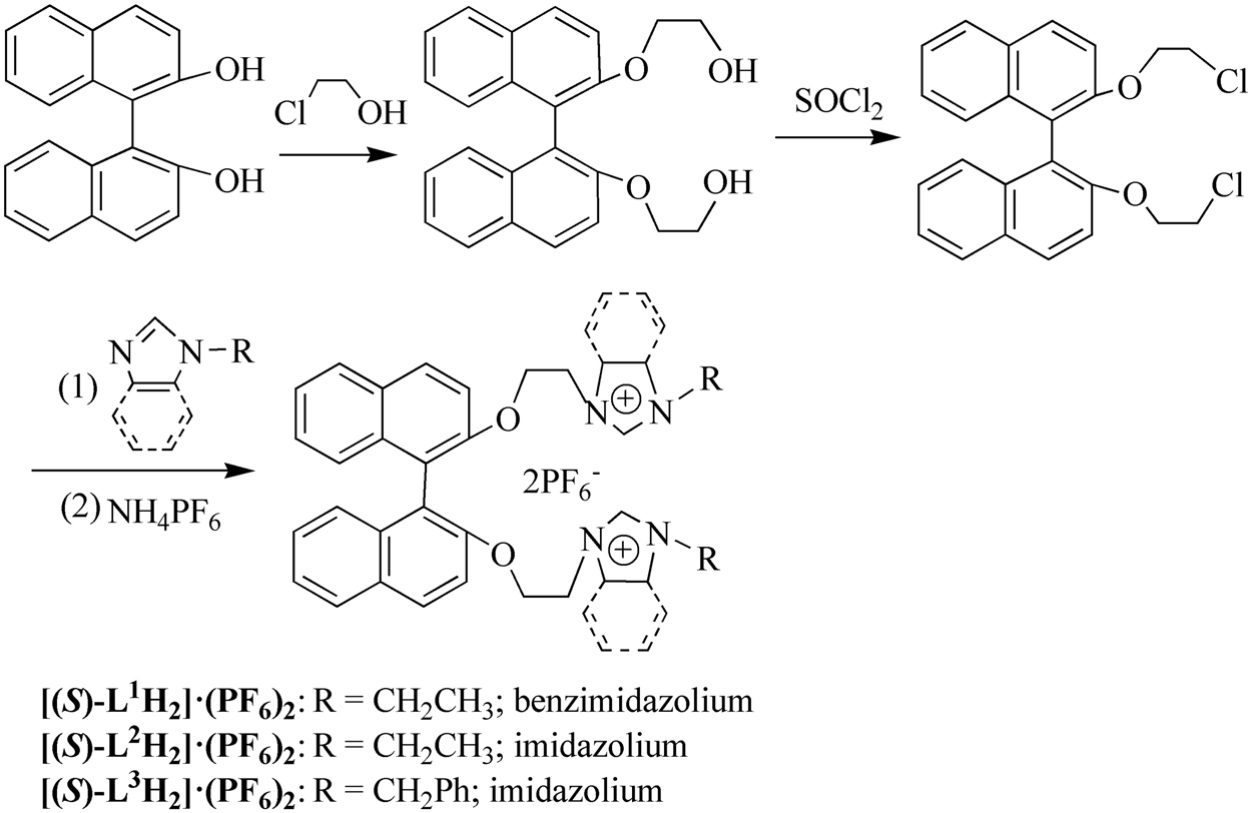
Preparation of precursors **[(** ***S*** **)-L** ^**1**^ **H**_**2**_ **]·(PF**_**6**_ **)**_**2**_ **-[(** ***S*** **)-L** ^**3**^ **H**_**2**_ **]·(PF**_**6**_ **)**_**2**_.

**Figure 2:**
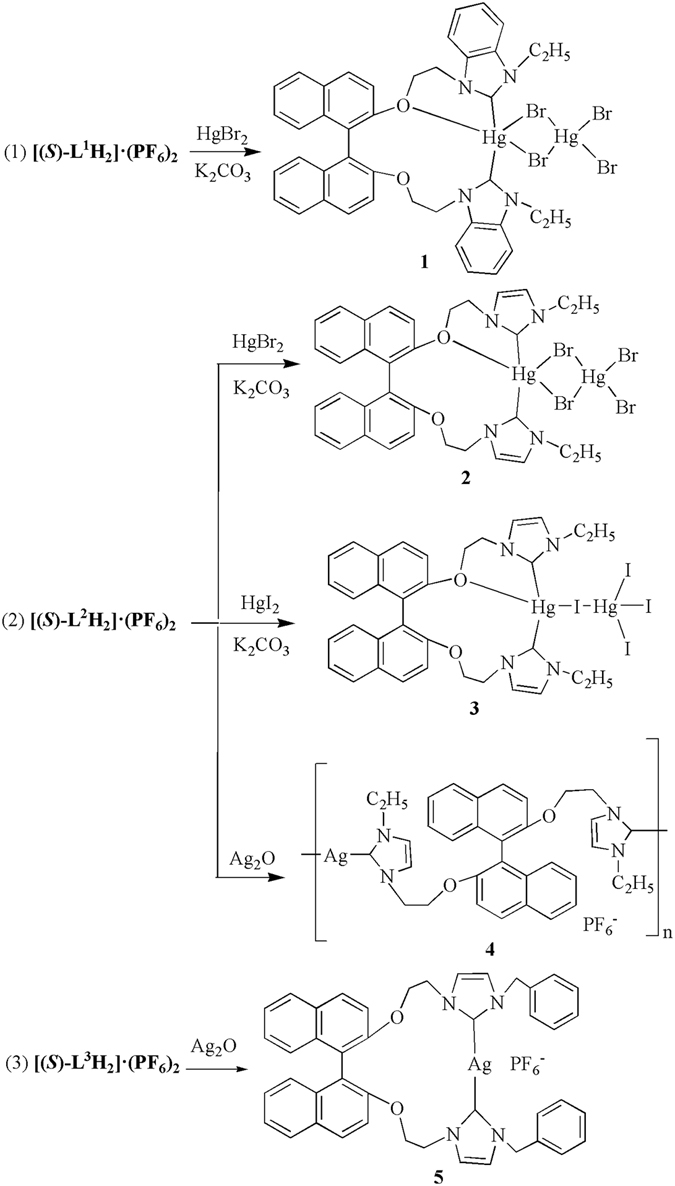
Preparation of complexes **1**–**5**.

As shown in Fig. 1, (S)-2,2′-dihydroxy-1,1′**-**binaphthyl as a starting material reacted with 2-chloroethanol to afford (*S*)-2,2′*-*di(2″-hydroxyethoxy)-1,1′-binaphthyl. Subsequent chlorination of hydroxyl groups with thionyl chloride generated (*S*)-2,2′*-*di(2″-chloroethoxy)-1,1′-binaphthyl. The reaction between (*S*)-2,2′*-*di(2″-chloroethoxy)-1,1′-binaphthyl and *N*-R-azole (R = ethyl or benzyl, azole = benzimidazole or imidazole) in toluene gave the bis-azolium salts [(***S***)**-L** ^**1**^ **H**_**2**_]**·Cl**_**2**_ ~ [(***S***)**-L** ^**3**^ **H**_**2**_]**·Cl**_**2**_. Synthesis of (*S*)-2,2′-bis[2″-(*N*-R-azoliumyl)ethoxy]-1,1′-binaphthyl hexafluorophosphate [(***S***)**-L** ^**1**^ **H**_**2**_]**·**(**PF**_**6**_)_**2**_ ~ [(**S**)**-L** ^**3**^ **H**_**2**_]**·**(**PF**_**6**_)_**2**_ was accomplished via anion exchange using ammonium hexafluorophosphate in methanol. Precursors of [(***S***)**-L** ^**1**^ **H**_**2**_]**·**(**PF**_**6**_)_**2**_ ~ [(***S***)**-L** ^**3**^ **H**_**2**_]**·**(**PF**_**6**_)_**2**_ are stable toward air and moisture, and soluble in organic solvents, such as CH_2_Cl_2_, CH_3_CN and DMSO, however, the solubility is poor in water, petroleum ether and diethyl ether. In the^1^H NMR spectra of [(***S***)**-L** ^**1**^ **H**_**2**_]**·**(**PF**_**6**_)_**2**_ ~ [(***S***)**-L** ^**3**^ **H**_**2**_]**·**(**PF**_**6**_)_**2**_, the proton signals (NC*H*N) of benzimidazolium (or imidazolium) appear at *δ* = 8.51–9.17 ppm, which are consistent with the chemical shifts of reported benzimidazolium (or imidazolium) salts^35–41^.

### Synthesis and characterization of complexes 1–5

As shown in Fig. 2, the reaction of [(***S***)**-L** ^**1**^ **H**_**2**_]·(**PF**_**6**_)_**2**_ or [(***S***)-**L** ^**2**^ **H**_**2**_]·(**PF**_**6**_)_**2**_ with HgBr_2_ or HgI_2_ in CH_3_CN/ClCH_2_CH_2_Cl or CH_3_CN/DMSO in the presence of anhydrous K_2_CO_3_ afforded complexes [(*S*)-L^1^Hg(HgBr_4_)] (**1**), [(*S*)-L^2^Hg(HgBr_4_)] (**2**) and [(*S*)-L^2^Hg(HgI_4_)] (**3**), respectively. Complex {[(*S*)-L^2^Ag](PF_6_)}_n_ (**4**) was prepared via the reaction of [(***S***)**-L** ^**2**^ **H**_**2**_]**·**(**PF**_**6**_)_**2**_ with Ag_2_O in acetonitrile. Similarly, complex [(*S*)-L^3^Ag](PF_6_) (**5**) could be obtained via the reaction of [(***S***)**-L** ^**3**^ **H**_**2**_]·(**PF**_**6**_)_**2**_ with Ag_2_O in ClCH_2_CH_2_Cl/DMSO.

The single crystals of complexes **1–5** suitable for X-ray diffraction were grown by slow diffusion of diethyl ether into their solutions. Complexes **1–5** demonstrate good stability to heat, air and moisture. They are soluble in CH_3_CN and DMSO, but almost insoluble in diethyl ether and hydrocarbon solvents. Complexes **4** and **5** are slightly light-sensitive in the solution, but inert in solid state. In^1^H NMR spectra of **1**–**5**, the resonances for the benzimidazolium (or imidazolium) protons (NC*H*N) disappear and the chemical shifts of other hydrogen atoms are similar to those of corresponding precursors. In^13^C NMR spectra, the signals for the carbene carbons of **1**–**3** are observed at *δ* = 174.3–175.0 ppm, which are similar to the known metal carbene complexes^42–49^. The signals for the carbene carbons in silver(I) complexes **4** and **5** are invisible. This phenomenon also has been reported for some silver(I)-carbene complexes, which may result from the fluxional behavior of the NHC silver(I) complexes^50–55^.

### Structures of precursor [(*S*)-L^2^H_2_]·(PF_6_)_2_ and complexes 1–5

**Figure 3:**
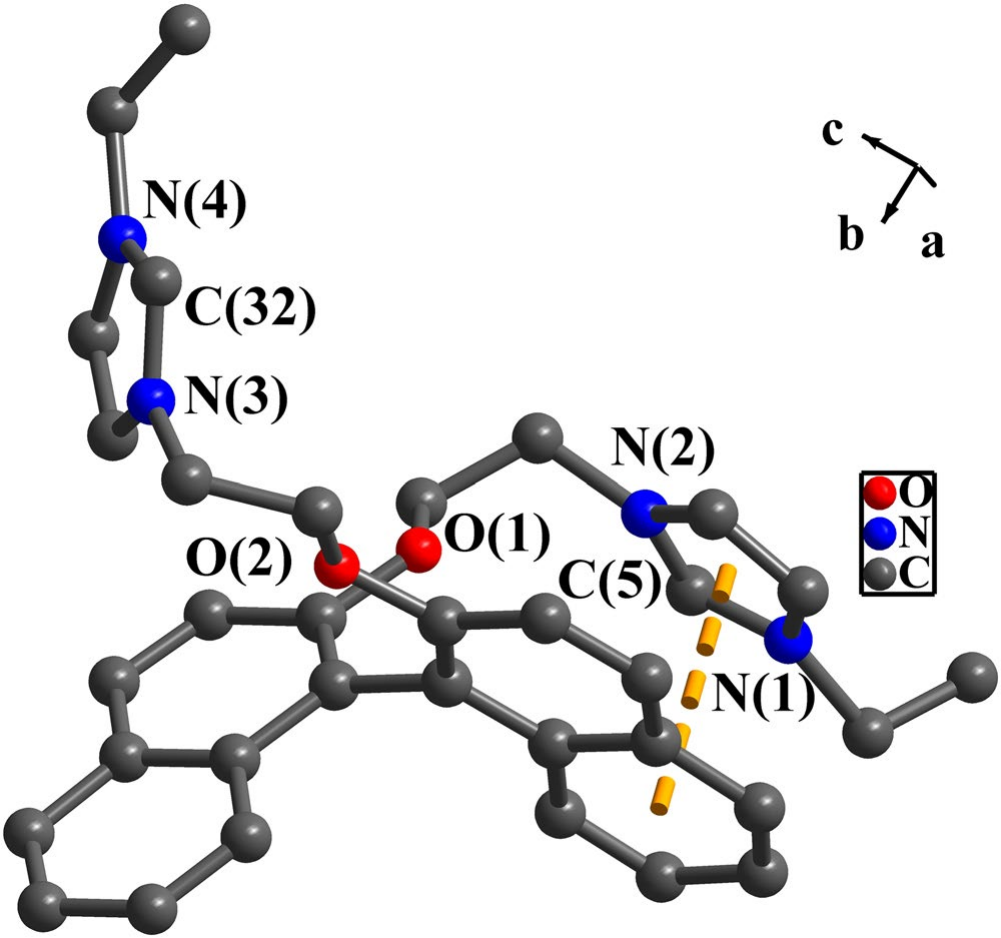
Perspective view of [(***S***)**-L** ^**2**^ **H**_**2**_]**·**(**PF**_**6**_)_**2**_. All hydrogen atoms were omitted for clarity. Selected bond lengths (Å) and angles (°): C(5)-N(1) 1.321(6), C(5)-N(2) 1.302(6); N(1)-C(5)-N(2) 108.7(4), N(3)-C(32)-N(4) 108.8(4).

**Figure 4:**
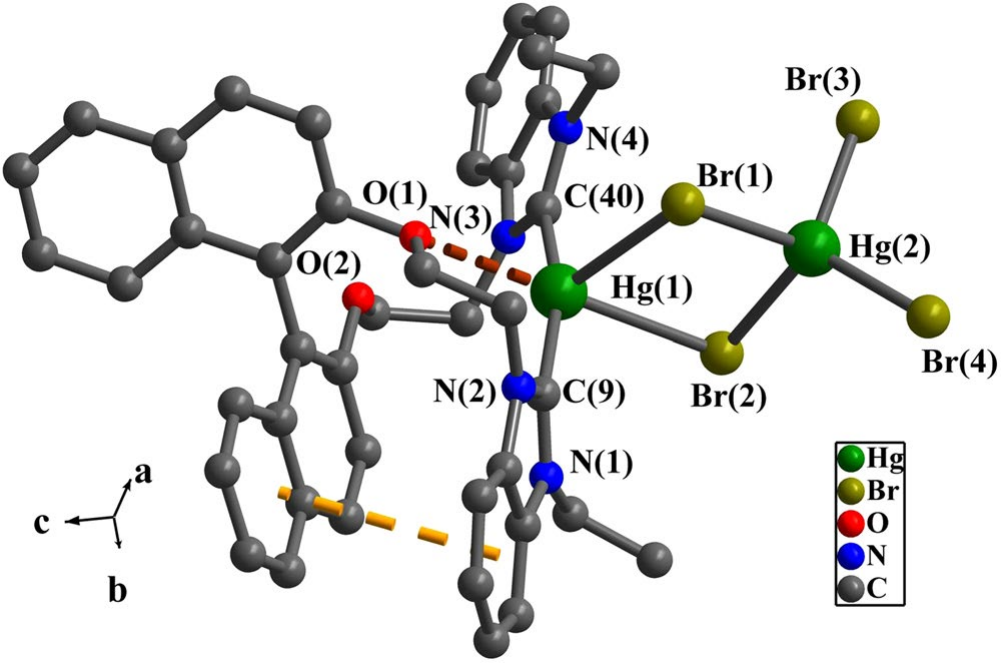
Perspective view of **1**. All hydrogen atoms were omitted for clarity. Selected bond lengths (Å) and angles (°): Hg(1)-Br(1) 3.037(1), Hg(1)-Br(2) 2.988(1), Hg(1)-C(9) 2.093(1), Hg(1)-C(40) 2.091(1), Hg(2)-Br(1) 2.662(1), Hg(2)-Br(2) 2.642(1), Hg(2)-Br(3) 2.562(1), Hg(2)-Br(4) 2.533(1); N(1)-C(9)-N(2) 108.4(9), N(3)-C(40)-N(4) 106.4(9), C(9)-Hg(1)-C(40) 162.2(4), C(40)-Hg(1)-Br(1) 105.8(3), Br(1)-Hg(2)-Br(2) 95.7(4).

**Figure 5:**
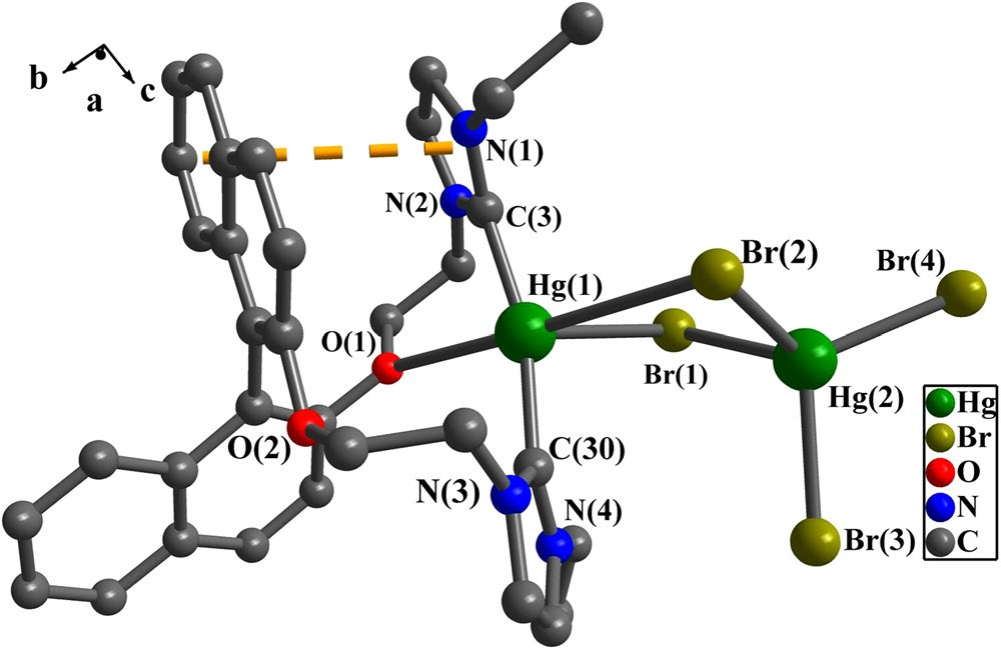
Perspective view of **2**. All hydrogen atoms were omitted for clarity. Selected bond lengths (Å) and angles (°): Hg(1)-C(3) 2.071(9), Hg(1)-C(30) 2.069(8), Hg(1)-Br(1) 3.073(1), Hg(2)-Br(1) 2.682(1), Hg(2)-Br(2) 2.638(1), Hg(2)-Br(3) 2.587(1), Hg(2)-Br(4) 2.568(1); N(3)-C(30)-N(4) 106.6(8), N(1)-C(3)-N(2) 105.9(8), C(3)-Hg(1)-C(30) 165.5(4), Br(1)-Hg(2)-Br(2) 97.4(3).

**Figure 6:**
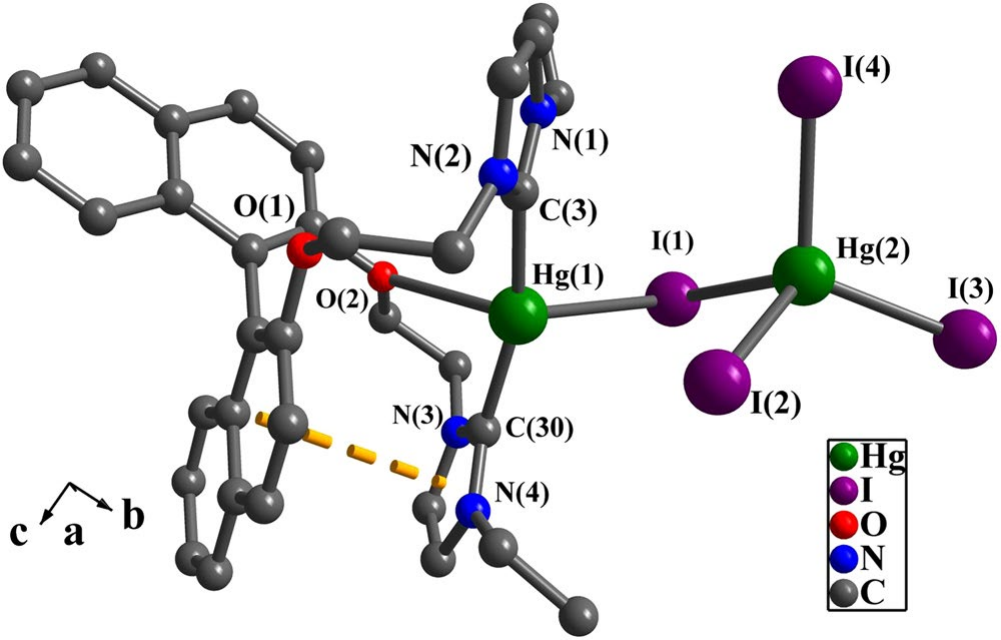
Perspective view of **3**. All hydrogen atoms were omitted for clarity. Selected bond lengths (Å) and angles (°): Hg(1)-I(1) 3.204(6), Hg(1)-C(30) 2.068(7), Hg(1)-C(3) 2.066(7), Hg(2)-I(1) 2.855(6), Hg(2)-I(2) 2.788(7), Hg(2)-I(3) 2.732(7), Hg(2)-I(4) 2.743(7); N(1)-C(3)-N(2) 105.9(6), N(3)-C(30)-N(4) 106.9(6), C(3)-Hg(1)-C(30) 164.6(3), C(30)-Hg(1)-I(1) 91.1(1), I(1)-Hg(2)-I(2) 98.6(2).

**Figure 7:**
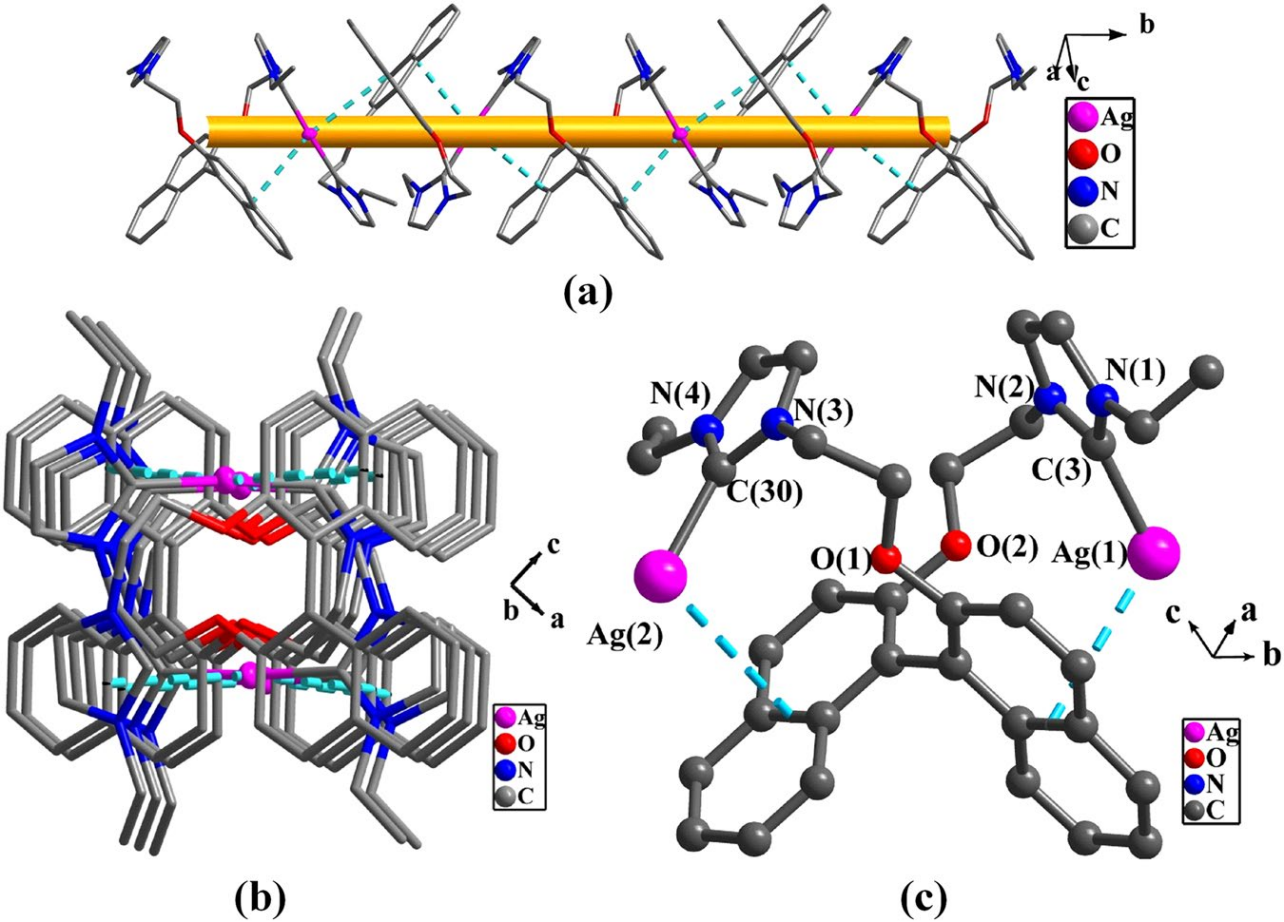
(**a**) 1D polymeric chains of complex **4**; (**b**) The view of *b* axis; (**c**) Perspective view of the monomer. All hydrogen atoms were omitted for clarity. Selected bond lengths (Å) and angles (°): C(3)-Ag(1) 2.084(6), C(30)-Ag(2) 2.081(6); N(3)-C(30)-N(4) 104.6(5), N(1)-C(3)-N(2) 104.5(5), C(3)-Ag(1)-C(30) 175.0(2).

**Figure 8:**
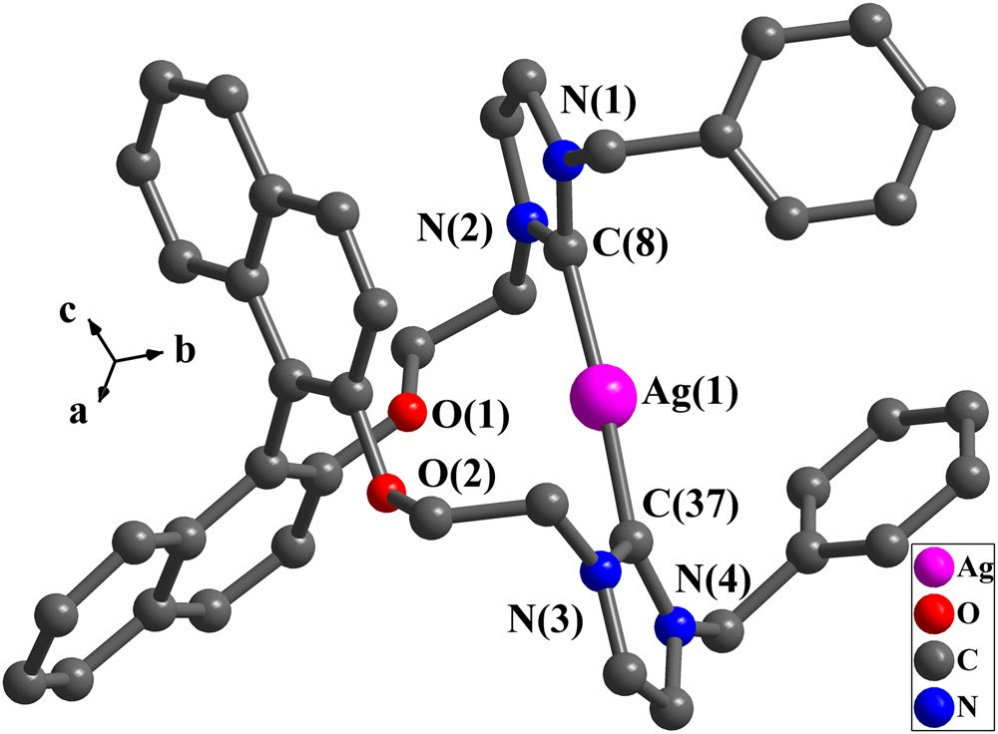
Perspective view of **5**. All hydrogen atoms were omitted for clarity. Selected bond lengths (Å) and angles (°): Ag(1)-C(8) 2.086(4), Ag(1)-C(37) 2.090(4); C(8)-Ag(1)-C(37) 173.4(1), N(2)-C(8)-N(1) 104.5(4), N(3)-C(37)-N(4) 104.5(3). All hydrogen atoms were omitted for clarity.

In the crystal structure of [(***S***)**-L** ^**2**^ **H**_**2**_]**·**(**PF**_**6**_)_**2**_ and complexes **1**–**5** (Figs 3–8), two naphthalene rings form the dihedral angles of 74.4(4)-82.5(6)°. The dihedral angles between two benzimidazole (or imidazole) rings vary from 31.1(1)° to 64.2(3)° (Table S1). The internal ring angles (N-C-N) at the carbene centers in complexes **1**–**5** are between 104.5(3)° and 108.4(9)°. These values are similar to those of known NHC-metal complexes^42–49^. In [(***S***)**-L** ^**2**^ **H**_**2**_]**·**(**PF**_**6**_)_**2**_ and complexes **1**–**3**, the intra-molecular π-π interactions between naphthalene rings and benzimidazole (or imidazole) rings are observed^56, 57^ (Table S2). In complexes **1**–**3**, one oxygen atom in each ligand participates in coordination with Hg(II) ion, and each of the molecules contains one 11-membered ring and one 6-membered ring. The distances of Hg-O are 3.061(7) Å for **1**, 2.837(5) Å for **2** and 2.861(5) Å for **3** respectively. These values are longer than normal distances of Hg-O (2.56–2.66 Å)^58^, but shorter than the sum of the van der Waals Radii between Hg(II) ion and oxygen atom (van der Waals Radii of mercury and oxygen being 1.70 Å and 1.40 Å). The bond angles of C-Hg-C are in the range of 162.2(4)°-165.5(4)°. The Hg-C bond distances are from 2.066(7) Å to 2.093(1) Å. Both are similar to the known NHC-Hg(II) complexes^59–63^. The long Hg(1)···Hg(2) separations in **1**–**3** (4.032(9) Å for **1**, 4.003(5) Å for **2** and 4.233(6) Å for **3**) suggest the nonexistence of metal-metal interactions.

In complex **1** or **2** (Figs 4 and 5), Hg(1) is penta-coordinated with two carbene carbon atoms, two bromine atoms and one oxygen atom to adopt a trigonal bipyramidal geometry. And Hg(2) is tetra-coordinated with four bromine atoms (two bridging bromine Br(1) and Br(2) and two terminal bromine Br(3) and Br(4)) to adopt a tetrahedral geometry. A distorted Hg_2_Br_2_ quadrangular geometry in **1** or **2** is formed by Hg(1), Br(1), Hg(2) and Br(2), in which the dihedral angles between Br(1)-Hg(1)-Br(2) plane and Br(1)-Hg(2)-Br(2) plane are 13.6(4)° for **1** and 36.4(2)° for **2**. The bond angles of Br-Hg(2)-Br are 95.7(4)-116.1(5)° for **1** and 97.4(3)-119.3(4)° for **2**. The bond angle of Br(2)-Hg(1)-O(1) for **1** is 166.1(1)° and 177.4(1)° for **2**. The distances of Hg(2) and terminal bromine for either **1** or **2** are between 2.533(1) Å and 2.587(1) Å, which are in the normal range of Hg-Br bond^64–66^. Notably, compared with the distances between the terminal bromine and mercury, the bridging bromine atoms have relatively longer bond distances of 2.638(1) Å-3.257(1) Å with mercury.

Other than complexes **1** and **2**, Hg(1) in **3** is tetra-coordinated with two carbene carbon atoms, one oxygen atom and one iodine atom (Fig. 6). Hg(2) is tetra-coordinated with four iodine atoms. Both Hg(1) and Hg(2) are connected together via the bridging iodine atom (I(1)). The bond angles of I-Hg(2)-I are from 98.6(2)° to 117.1(2)°, and Hg(2)-I_terminal_ distances are from 2.732(7) Å to 2.855(6) Å. These values fall in the normal range^58^. The distance of Hg(1)-I(1) (3.204(6) Å) is longer than aforementioned normal values.

Crystal structure analysis of **4** reveals the formation of 1D helical polymeric chain via NHC ligand (***S***)**-L** ^**2**^ and silver(I) ion (Fig. 7(a)), in which Ag-π interactions are observed (π system being from naphthalene ring, and the separation of Ag-π being 3.591(1) Å)^67, 68^. There exists a cavity of about 3.35 Å × 3.60 Å in the center of the chain by viewing from *b* axis (Fig. 7(b)). The distance between adjacent two silver(I) ions in the chain is 8.177(4) Å. The coordination geometry of each silver(I) ion is approximately linear with 175.0(2)° angle of C(3)-Ag(1)-C(30) and 2.081(6) Å-2.084(6) Å bond distance of Ag-C (Fig. 7(c)). Similar observations were also reported for known NHC silver(I) complexes^69^.

In complex **5** (Fig. 8), one 15-membered macrometallocycle is formed by one ligand (***S***)**-L** ^**3**^ and one silver(I) ion, in which silver(I) ion is di-coordinated with two carbene carbon atoms to adopt an approximately linear geometry. The bond angle of C(8)-Ag(1)-C(37) is 173.4(1)°. The two Ag-C bond distances are 2.086(4) Å and 2.090(4) Å respectively. Ag···O separation of 3.2 Å is longer than the sum of the van der Waals Radii between Ag(I) ion and oxygen atom (3.1 Å) which indicates the absence of Ag···O interactions.

### Powder X-ray diffraction

In order to establish their crystalline phase purity, powder X-ray diffraction (PXRD) experiments were carried out on complexes **1–5**. As shown in the PXRD patterns (Figure S1–S5), the excellent agreement between the experimental PXRD patterns of the bulk samples **1–5** and the patterns simulated from the single-crystal data proved the crystalline phase purity of the corresponding **1–5**.

### Thermogravimetric analysis of complexes 1–5

To examine the thermal stability of complexes **1**–**5**, the thermogravimetric analyses for crystal samples of **1**–**5** were performed under a simulated air atmosphere with a heating rate of 20 °C min^−1^ from ambient temperature up to 600 °C. As demonstrated in Figures S6 and S7, the TG curves of **1** and **2** revealed that complex **1** started to decompose from ambient temperature to 172.4 °C and complex **2** started to decompose from ambient temperature to 243.5 °C. The curves represented the losses of approximately 0.5 equiv. of solvent molecule (ClCH_2_CH_2_Cl) for complex **1** (calcd: 3.53%, found: 3.50%) and 1.5 equiv. of solvent molecules (DMSO) for complex **2** (calcd: 8.57%, found: 8.56%). 49.29% weight loss from 172.4 °C to 434.3 °C for complex **1** and 55.35% weight loss from 243.5 °C to 405.6 °C for complex **2** were experienced, which resulted from the thermal decomposition of the organic components and did not stop until the heating ended at 600 °C. From the TG curve of **3** (Figure S8), it has been found that this compound decomposed from ambient temperature to 211.4 °C, which represented the loss of approximately 1 equiv. of solvent molecule (DMSO) (calcd: 5.13%, found: 5.12%). Further decomposition from 211.4 °C to 281.5 °C represented the loss 2 equiv. of iodide atoms (calcd: 16.73%, found: 16.78%). Complex **3** experienced weight loss of 45.61% from 281.5 °C to 402.4 °C due to the thermal decomposition of the organic components. It did not stop until heating ended at 600 °C. The TG curve depicted in Figure S9 indicated that complex **4** had a high thermal stability which remained unchanged up to 241.6 °C. Almost one-step weight loss of 56.05% was detected from 241.6 °C to 395.6 °C, which was attributed to the thermal decomposition of the organic components and did not stop until heating ends at 600 °C. As shown in Figure S10, compound **5** started to decompose from ambient temperature to 127.3 °C, which represented the loss of approximately 0.5 equiv. of solvent molecule (DMSO) (calcd: 4.12%, found: 4.20%). With temperature increasing to 165.1 °C from 127.3 °C, weight loss of 11.40% represented the loss of 1 equiv. of silver ion (calcd: 11.39%). This compound experienced weight loss of 25.29% from 165.1 °C to 436.4 °C, which was attributed to the thermal decomposition of the organic components and did not stop until heating ended at 600 °C.

### IR spectral analysis of [(*S*)-L^1^H_2_]·(PF_6_)_2_ ~ [(*S*)-L^3^H_2_]·(PF_6_)_2_ and complexes 1–5

In the infrared spectra of precursors [(***S***)**-L** ^**1**^ **H**_**2**_]**·**(**PF**_**6**_)_**2**_ ~ [(***S***)**-L** ^**3**^ **H**_**2**_]**·**(**PF**_**6**_)_**2**_ and complexes **1**–**5** (Figures S11–S15), the absorption bands around 3000 cm^−1^ can be assigned to *ν*(C-H) modes. The absorption bands in the region of 1598–1590 cm^−1^ may result from *ν*(C = N) of benzimidazole or imidazole rings. The absorption bands in the regions of 1271–1218 cm^−1^ and 1087–1054 cm^−1^ may be ascribed to the *ν*( = C-O-C) moiety. In [(***S***)**-L** ^**1**^ **H**_**2**_]**·**(**PF**_**6**_)_**2**_ ~ [(***S***)**-L** ^**3**^ **H**_**2**_]**·**(**PF**_**6**_)_**2**_, complexes **4** and **5** containing hexafluorophosphate anion, the absorption bands at about 840 cm^−1^ and 550 cm^−1^ originate from P-F stretching vibration and P-F flexural vibration, respectively. These values are consistent with reported results in literatures^70^. By contrast, no obvious absorption bands at about 840 cm^−1^ and 550 cm^−1^ are observed for complexes **1**–**3** due to the absence of hexafluorophosphate anion.

### Recognition of H_2_PO_4_^−^ using complex 5 as a receptor

The screening experiment of some anions (F^−^, Cl^−^, Br^−^, I^−^, H_2_PO_4_^−^, HSO_4_^−^, OAc^−^ and NO_3_^−^, use of their TBA^+^ salts) using complexes **1**–**5** as hosts were carried out via fluorescence spectroscopy in acetonitrile at 25 °C. The addition of anions to the solutions of **1**–**4** did not lead to obvious fluorescence intensities change. Upon the addition of H_2_PO_4_^−^ to the solution of **5**, the fluorescence intensity of **5** remarkably decreased while other anions did not exhibit evident influence on the fluorescence intensity of **5**. Therefore, complex **5** was selected for the anions recognition performance investigation.

**Figure 9:**
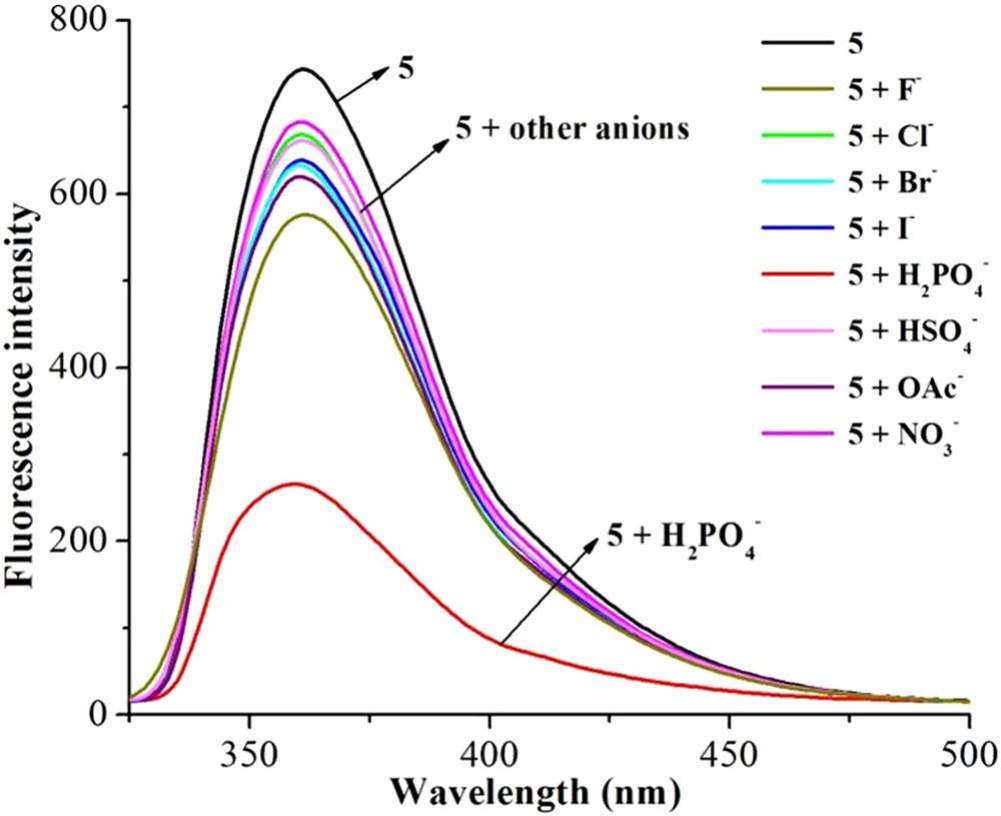
Fluorescence spectra of **5** (1 × 10^−5^ mol/L) and upon the addition of salts (20.0 equiv) of F^−^, Cl^−^, Br^−^, I^−^, H_2_PO_4_^−^, HSO_4_^−^, OAc^−^ and NO_3_^−^ in CH_3_CN at 25 °C.

**Figure 10:**
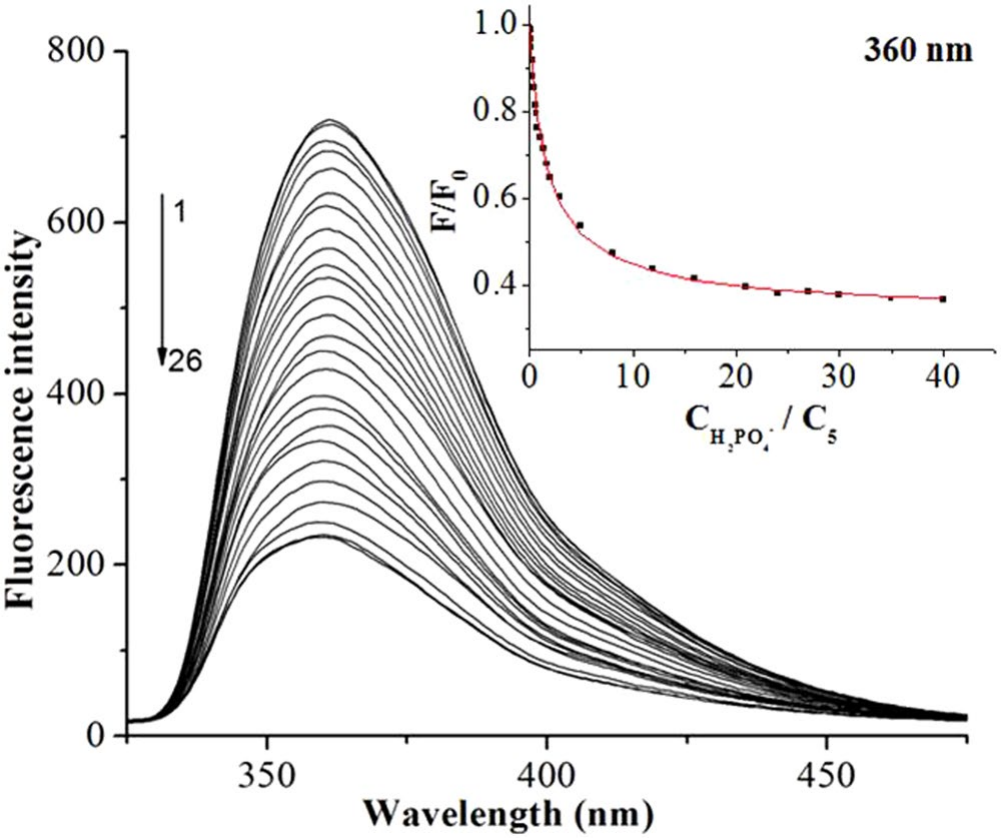
Fluorescence titration spectra of **5** (1.0 × 10^−5^ mol/L) in the presence of different concentrations of H_2_PO_4_^−^ in CH_3_CN at 25 °C. C_H2PO4_^−^ for curves 1–26 (from top to bottom) are 0, 0.01, 0.07, 0.11, 0.17, 0.25, 0.33, 0.43, 0.55, 0.67, 0.8, 1, 1.35, 1.75, 2, 3, 5, 8, 12, 16, 21, 24, 27, 30, 35, 40 × 10^−5^ mol/L (λ_ex_ = 280 nm). Inset: the fluorescence at 360 nm of **5** as a function of H_2_PO_4_^−^ concentration.

As illustrated in Fig. 9, the receptor **5** (1 × 10^−5^ mol/L) exhibited a strong emission peak at 360 nm, which was attributed to the emission of binaphthyl (λ_ex_ = 280 nm, the excitation and emission slits: 3 nm and 1.5 nm). Upon the addition of 20 equiv. of F^−^, Cl^−^, Br^−^, I^−^, HSO_4_^−^, OAc^−^ or NO_3_^−^, the fluorescence emission of **5** had a slight decrease. However, the addition of same amount of H_2_PO_4_^−^ caused a remarkable decrease of the fluorescence emission of **5**. This phenomenon might be attributed to the switch-on of the photo-induced electron transfer (PET) process from the imidazole ring to the binaphthyl in the presence of H_2_PO_4_^−^^20, 71–73^. In UV/vis experiment (Figure S16), the receptor **5** (1 × 10^−5^ mol/L) exhibited an absorption peak at around 220–245 nm in acetonitrile at 25 °C which originated from the *E*_1_ absorption band of binaphthyl. With the addition of H_2_PO_4_^−^, the absorption peak of **5** at 220–245 nm decreased obviously. It can be concluded that **5** had the ability to selectively discriminate H_2_PO_4_^−^ from other anions. Figure 10 demonstrated the fluorescence spectra of **5** (1 × 10^−5^ mol/L) in the presence of different amounts of H_2_PO_4_^−^, in which the fluorescence intensities at 360 nm decreased gradually with the increasing concentration of H_2_PO_4_^−^. In the inset of Fig. 10, when the ratio of C_H2PO4_^−^/C_**5**_ was no more than 8:1, the fluorescence intensity remarkably decreased with the enhancement of H_2_PO_4_^−^ concentration. Changed the ratio from 8:1 to 24:1, the decreasing tendency of the fluorescence intensity slowed down. When the ratio exceeded 40:1, higher C_H2PO4_^−^ did not lead to further decrease of emission. The stability constant *Ks* for **5**·H_2_PO_4_^−^ was calculated as 1.03 × 10^5^ M^−1^ (R = 0.991) by using the nonlinear least-square analysis^74, 75^:

$$\begin{array}{rcl}F/{F}_{0} & = & 1+({F}_{\max }/2{F}_{0}-1/2)\{1+{{\rm{C}}}_{{{\rm{H}}}_{{\rm{2}}}{{\rm{PO}}}_{{\rm{4}}}}^{-}{/C}_{{\bf{5}}}\\  &  & +1/Ks{{\rm{C}}}_{{\bf{5}}}-[1+{{\rm{C}}}_{{{\rm{H}}}_{{\rm{2}}}{{\rm{PO}}}_{{\rm{4}}}}^{-}{/C}_{5}\\  &  & +1/Ks{C}_{{\bf{5}}})2+1+{{\rm{C}}}_{{{\rm{H}}}_{{\rm{2}}}{{\rm{PO}}}_{{\rm{4}}}}^{-}{/C}_{{\bf{5}}}{]}^{1/2}\}\end{array}$$

where *F* and *F*_0_ are the fluorescence intensity of **5** in the presence and absence of H_2_PO_4_^−^; *F*_max_ is the fluorescence intensity in the maximum concentration of H_2_PO_4_^−^; CH_2_PO_4_^−^ and C_**5**_ are the concentrations of H_2_PO_4_^−^ and **5**, respectively; *Ks* is the stability constant. From the changes in H_2_PO_4_^−^ dependent fluorescence intensity (Figure S17), the detection limit was estimated to be 4.9 × 10^−8^ mol/L for **5** ^76^.

**Figure 11:**
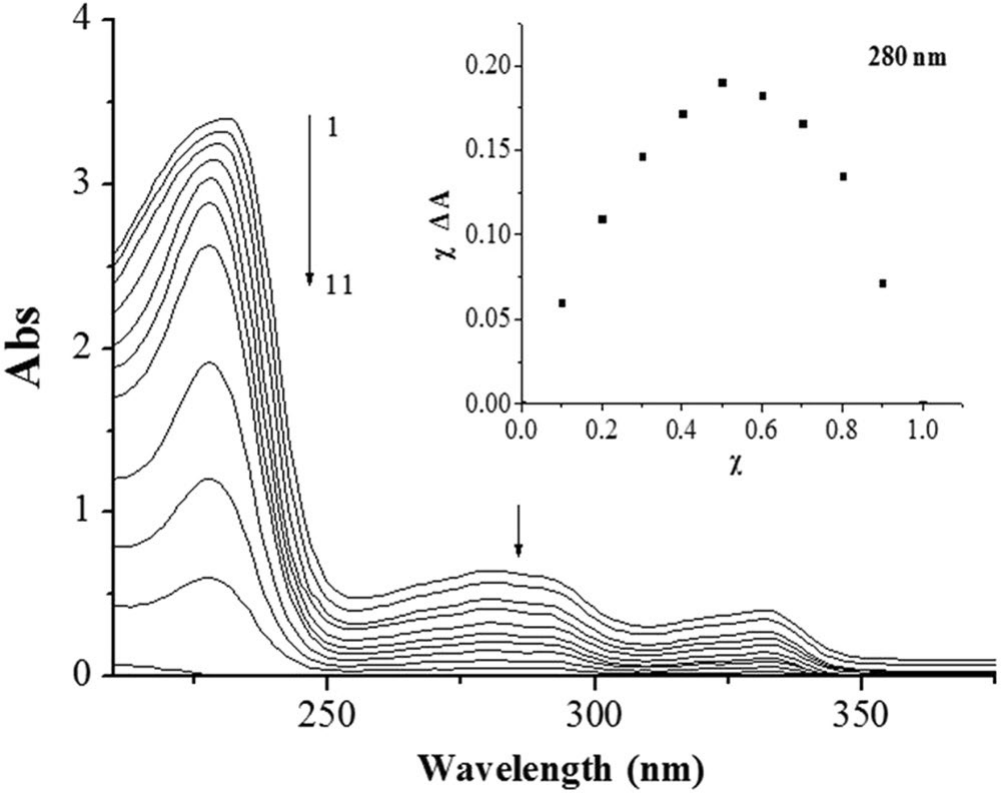
UV/vis absorption spectra of **5** (6.0 × 10^−5^ mol/L) in CH_3_CN at 25 °C. The concentrations of H_2_PO_4_^−^ for curves 1–11 (from bottom to top) are: 0, 0.6, 1.2, 1.8, 2.4, 3.0, 3.6, 4.2, 4.8, 5.4, 6.0 × 10^−5^ mol/L. Inset: The Job’s plot for **5**·H_2_PO_4_^−^ complex in CH_3_CN at 280 nm.

In UV/vis titration experiment (Figure 11), the UV/vis absorption spectra of **5** dropped gradually with the increase of the molar fraction of H_2_PO_4_^−^. It was notable that a 1:1 complexation stoichiometry for **5**·H_2_PO_4_^−^ was established by Job’s plot analysis at 280 nm (inset of Fig. 11) ^77, 78^, where the products (*χ*Δ*A*) between molar fractions and the discrepancy of the absorption bands were plotted against molar fractions (*χ*) of **5** under the conditions of a constant total concentration. When the molar fraction of **5** was 0.5, the *χ*Δ*A* value for **5**·H_2_PO_4_^−^ reached maximum^79^.

According to the size of the cavity (8.4 Å × 6.7 Å) and structural characteristics of **5**, the size of H_2_PO_4_^−^ (radius of H_2_PO_4_^−^ being *ca*. 2.9 Å) is able to match well with that of **5**. Possible binding sites in **5** contain oxygen atoms and silver(I) ion. The acting force between H_2_PO_4_^−^ and **5** might be the result of combined effects of several weak intermolecular interactions, such as O-H···O hydrogen bonds and Ag···O interactions. But no significant changes of proton signals were observed in terms of the^1^H NMR spectra of **5** and **5**·H_2_PO_4_^−^.

To further explore the utility of **5** as a selective fluorescence receptor for H_2_PO_4_^−^, the competition experiments were conducted, where **5** (1 × 10^−5^ mol/L) was firstly mixed with 10 equiv. of various anions (F^−^, Cl^−^, Br^−^, I^−^, HSO_4_^−^, OAc^−^ and NO_3_^−^), and then 10 equiv. of H_2_PO_4_^−^ was added. As displayed in Figure S18, no obvious interference was observed in the presence of 10 equiv. of various anions. In high resolution mass spectrometry (HRMS) analysis of **5**·H_2_PO_4_^−^ (Figure S19), m/z (859.2) was observed which provided additional evidence for the formation of a 1:1 complex between **5** and H_2_PO_4_^−^. This result was consistent with the findings of the Job’s plot analysis (inset of Fig. 11).

## Conclusion

In summary, three bis-azolium salts [(***S***)**-L** ^**1**^ **H**_**2**_]**·**(**PF**_**6**_)_**2**_ ~ [(***S***)**-L** ^**3**^ **H**_**2**_]**·**(**PF**_**6**_)_**2**_ and their five NHC Hg(II) and Ag(I) complexes **1**–**5** have been prepared and characterized. In complexes **1**–**3**, each molecule contains one 11-membered ring and one 6-membered ring. 1D helical polymeric chain of **4** is formed via ligand **(** ***S***)**-L** ^**2**^ and silver(I) ion. One 15-membered macrometallocycle of **5** is formed by one ligand (***S***)**-L** ^**3**^ and one silver(I) ion. Interestingly, the macrometallocycle **5** is found to be high selectivity and sensitivity for H_2_PO_4_^−^ detection. This indicates that **5** can distinguish H_2_PO_4_^−^ from other anions by using the methods of fluorescence and UV/vis spectroscopy. Even if the detection limit is below 4.9 × 10^−8^ mol/L, the receptor **5** for H_2_PO_4_^−^ detection is still sensitive. This character of complex **5** makes it possible to be applied in environmental science and life science. Further studies of new organometallic complexes from precursor [(***S***)**-L** ^**1**^ **H**_**2**_]**·**(**PF**_**6**_)_**2**_ ~ [(***S***)**-L** ^**3**^ **H**_**2**_]**·**(**PF**_**6**_)_**2**_ as well as analogous ligands are underway.

## Experimental

### General procedures

All the reagents for synthesis and analyses were of analytical grade and used without further purification. Melting points were determined on an Digital Vision MP Instrument.^1^H and^13^C NMR spectra were recorded at 400 MHz and 100 MHz, respectively. Chemical shifts, *δ*, were reported in ppm relative to the internal standard TMS for both^1^H and^13^C NMR. *J* values were given in Hz. The elemental analyses of all compounds were obtained from the powder compounds recrystallised. The fluorescence spectra were performed using a Cary Eclipse fluorescence spectrophotometer. UV-vis spectra were recorded on a JASCO-V570 spectrometer. EI mass spectra were recorded on a VG ZAB-HS mass spectrometer (VG, U.K.). The powder X-ray diffractometry (PXRD) study was performed on a PANalytical X-Pert Pro diffractometer with Cu-Kα radiation. The thermogravimetric analysis (TGA) was performed with a NETZSCH STA 449 C instrument. IR spectra (KBr) were taken on an Bruker Equinox 55 spectrometer.

### Preparation of (*S*)-2,2′*-*di(2″-hydroxyethoxy)-1,1′-binaphthyl

A suspension of (*S*)-2,2′*-*dihydroxy-1,1′**-**binaphthyl (5.000 g, 17.4 mmol) and potassium carbonate (9.646 g, 69.9 mmol) in DMF (100 mL) was stirred for 1 h at 50 °C. Then 2-chloroethanol (5.628 g, 69.9 mmol) was added dropwise and stirring was continued for 24 h under refluxing. The solvent was removed under vacuum and then 500 mL water was added. The solution was extracted with CH_2_Cl_2_ (3 × 30 mL) and the organic phase was dried over anhydrous MgSO_4_. After removing CH_2_Cl_2_, a pale yellow oil was obtained, which was recrystallized with MeOH to give (*S*)-2,2′*-*di(2″-hydroxyethoxy)-1,1′-binaphthyl as a white powder. Yield: 6.008 g (92%). M.p.: 112–114 °C. Anal. Calcd for C_24_H_22_O_4_: C, 76.98; H, 5.92%. Found: C, 76.63; H, 5.88%.^1^H NMR (400 MHz, CDCl_3_): *δ* 8.02 (d, *J* = 8.8 Hz, 2 H, Ar*H*), 7.93 (d, *J* = 8.0 Hz, 2 H, Ar*H*), 7.48 (d, *J* = 9.2 Hz, 2 H, Ar*H*), 7.41 (t, *J* = 7.4 Hz, 2 H, Ar*H*), 7.30 (t, *J* = 7.4 Hz, 2 H, Ar*H*), 7.17 (d, *J* = 8.4 Hz, 2 H, Ar*H*), 4.28 (m, 2 H, C*H*_2_), 4.08 (m, 2 H, C*H*_2_), 3.66 (m, 4 H, C*H*_2_), 2.31 (s, 2 H, O*H*).^13^C NMR (100 MHz, CDCl_3_): *δ* 153.5 (Ar*C*), 133.8 (Ar*C*), 129.8 (Ar*C*), 129.6 (Ar*C*), 128.1 (Ar*C*), 126.7 (Ar*C*), 125.2 (Ar*C*), 124.2 (Ar*C*), 120.3 (Ar*C*), 115.9 (Ar*C*), 71.7 (O*C*H_2_CH_2_), 61.2 (OCH_2_ *C*H_2_).

### Preparation of (*S*)-2,2′*-*di(2″-chloroethoxy)-1,1′-binaphthyl

To a chloroform (100 mL) solution of (*S*)-2,2′*-*di(2″-hydroxyethoxy)-1,1′-binaphthyl (5.000 g, 13.4 mmol) and pyridine (5.288 g, 66.8 mmol), thionyl chloride (7.947 g, 66.8 mmol) was added dropwise at room temperature within 1 h. Then the solution was stirred for 24 h at 70 °C. The mixture was cooled and washed with water (3 × 100 mL). The organic layer was dried over anhydrous MgSO_4_. After removing chloroform, (*S*)-2,2′*-*di(2″-chloroethoxy)-1,1′-binaphthyl was obtained as a pale yellow powder. Yield: 3.571 g (65%). M.p.: 101–103 °C. Anal. Calcd for C_24_H_20_O_2_Cl_2_: C, 70.08; H, 4.90%. Found: C, 70.37; H, 5.10%.^1^H NMR (400 MHz, CDCl_3_): *δ* 7.97 (d, *J* = 8.8 Hz, 2 H, Ar*H*), 7.88 (d, *J* = 8.4 Hz, 2 H, Ar*H*), 7.43 (d, *J* = 8.8 Hz, 2 H, Ar*H*), 7.37 (m, 2 H, Ar*H*), 7.23 (d, *J* = 1.2 Hz, 2 H, Ar*H*), 7.13 (d, *J* = 8.4 Hz, 2 H, Ar*H*), 4.22 (m, 4 H, C*H*_2_), 3.40 (t, *J* = 6.2 Hz, 4 H, C*H*_2_).^13^C NMR (100 MHz, CDCl_3_): *δ* 153.6 (Ar*C*), 134.0 (Ar*C*), 129.6 (Ar*C*), 127.9 (Ar*C*), 126.5 (Ar*C*), 125.4 (Ar*C*), 124.1 (Ar*C*), 121.0 (Ar*C*), 116.3 (Ar*C*), 70.1 (O*C*H_2_CH_2_), 41.7 (OCH_2_ *C*H_2_).

### Preparation of [(*S*)-L^1^H_2_]·(PF_6_)_2_

A solution of *N*-ethyl-benzimidazole (0.894 g, 6.1 mmol) and (*S*)-2,2′*-*di(2″-chloroethoxy)-1,1′-binaphthyl (1.000 g, 2.4 mmol) in toluene (35 mL)was stirred for 7 days under refluxing and a brown oil of (*S*)-2,2′-bis[2″-(*N*-ethyl-benzimidazoliumyl)ethoxy]-1,1′-binaphthyl chloride was formed. Then NH_4_PF_6_ (0.938 g, 5.7 mmol) was added to a methanol solution (100 mL) of (*S*)-2,2′-bis[2″-(*N*-ethyl-benzimidazolimyl)ethoxy]-1,1′-binaphthyl chloride (2.016 g, 2.4 mmol) with stirring for 3 days. A white precipitate was formed that was collected by filtration and washed with a small portion of methanol to give [(***S***)**-L** ^**1**^ **H**_**2**_]**·**(**PF**_**6**_)_**2**_. Yield: 1.906 g (85%). M.p.: 132–134 °C. Anal. Calcd for C_42_H_40_F_12_N_4_O_2_P_2_: C, 54.67; H, 4.37; N, 6.07%. Found: C, 54.42; H, 4.53; N, 6.34%.^1^H NMR (400 MHz, DMSO*-d*_6_): *δ* 9.17 (s, 2 H, 2-bimi*H*), 7.93 (m, 6 H, Ar*H*), 7.58 (t, *J* = 7.6 Hz, 2 H, Ar*H*), 7.36 (t, *J* = 4.6 Hz, 4 H, Ar*H*), 7.29 (m, 4 H, Ar*H*), 6.96 (t, *J* = 7.4 Hz, 2 H, Ar*H*), 6.52 (d, *J* = 8.4 Hz, 2 H, Ar*H*), 4.53 (s, 3 H, C*H*_2_), 4.36 (m, 3 H, C*H*_2_), 4.21 (t, *J* = 7.0 Hz, 3 H, C*H*_2_), 4.16 (t, *J* = 5.4 Hz, 3 H, C*H*_2_), 1.34 (t, *J* = 7.2 Hz, 6 H, C*H*_3_).^13^C NMR (100 MHz, DMSO*-d*_6_): *δ* 153.0 (Ar*C*), 141.6 (Ar*C*), 132.8 (Ar*C*), 131.2 (Ar*C*), 130.7 (Ar*C*), 129.9 (Ar*C*), 129.2 (Ar*C*), 128.4 (Ar*C*), 126.7 (Ar*C*), 126.6 (Ar*C*), 124.4 (Ar*C*), 119.5 (Ar*C*), 116.0 (Ar*C*), 113.8 (Ar*C*), 113.5 (Ar*C*), 67.5 (O*C*H_2_CH_2_), 49.0 (OCH_2_ *C*H_2_), 42.4 (*C*H_2_CH_3_), 14.2 (*C*H_3_) (bimi = benzimidazole). IR (KBr, cm^−1^): 3150w, 1591w, 1356w, 1159 s, 1074 s, 950 m, 840 s, 550 m.

### Preparation of [(*S*)-L^2^H_2_]·(PF_6_)_2_

This compound was prepared in an analogous manner to that of [(***S***)**-L** ^**1**^ **H**_**2**_]**·**(**PF**_**6**_)_**2**_, only *N*-ethyl-imidazole (0.584 g, 6.1 mmol) was used instead of *N*-ethyl-benzimidazole. Yield: 1.781 g (89%). M.p.: 124–126 °C. Anal. Calcd for C_34_H_36_F_12_N_4_O_2_P_2_: C, 49.64; H, 4.41; N, 6.81%. Found: C, 49.83; H, 4.54; N, 6.63%.^1^H NMR (400 MHz, DMSO*-d*_6_): *δ* 8.51 (s, 2 H, 2-imi*H*), 8.14 (d, *J* = 9.2, 2 H, Ar*H*), 8.02 (d, *J* = 8.0 Hz, 2 H, Ar*H*), 7.62 (d, *J* = 8.8 Hz, 2 H, Ar*H*), 7.40 (t, *J* = 7.4 Hz, 2 H, Ar*H*), 7.26 (s, 2 H, Ar*H*), 7.22 (t, *J* = 7.6 Hz, 2 H, Ar*H*), 6.78 (d, *J* = 7.2 Hz, 4 H, Ar*H*), 4.40 (q, *J* = 4.5 Hz, 4 H, C*H*_2_), 4.33 (q, *J* = 7.6 Hz, 4 H, C*H*_2_), 3.93 (q, *J* = 7.2 Hz, 4 H, C*H*_2_), 1.27 (t, *J* = 7.2 Hz, 6 H, C*H*_3_).^13^C NMR (100 MHz, DMSO*-d*_6_): *δ* 152.6 (Ar*C*), 135.1 (Ar*C*), 132.9 (Ar*C*), 129.6 (Ar*C*), 128.9 (Ar*C*), 128.0 (Ar*C*), 126.6 (Ar*C*), 124.2 (Ar*C*), 123.9 (Ar*C*), 121.7 (O*C*H_2_), 121.2 (Ph*C*), 118.7 (Ph*C*), 115.1 (Ph*C*), 68.6 (O*C*H_2_CH_2_), 48.3 (OCH_2_ *C*H_2_), 44.0 (*C*H_2_CH_3_), 14.8 (*C*H_3_) (imi = imidazole). IR (KBr, cm^−1^): 3162 m, 1591w, 1244w, 1087 m, 983w, 840 s, 551 m.

### Preparation of [(*S*)-L^3^H_2_]·(PF_6_)_2_

This compound was prepared in an analogous manner to that of [(***S***)**-L** ^**1**^ **H**_**2**_]**·**(**PF**_**6**_)_**2**_, only *N*-benzyl-imidazole (0.964 g, 6.1 mmol) was used instead of *N*-ethyl-benzimidazole. Yield: 1.954 g (86%). M.p.: 148–150 °C. Anal. Calcd for C_44_H_40_O_2_N_4_P_2_F_12_: C, 55.82; H, 4.26; N, 5.92%. Found: C, 55.63; H, 4.42; N, 5.77%. ^1^H NMR (400 MHz, DMSO*-d*_6_): *δ* 8.74 (s, 2 H, 2-imi*H*), 8.15 (d, *J* = 8.0 Hz, 2 H, Ar*H*), 8.03 (d, *J* = 2.0 Hz, 2 H, Ar*H*), 7.59 (d, *J* = 8.8 Hz, 2 H, Ar*H*), 7.42 (m, 8 H, Ar*H*), 7.30 (q, *J* = 5.1 Hz, 6 H, Ar*H*), 7.19 (t, *J* = 7.4 Hz, 2 H, Ar*H*), 6.79 (d, *J* = 8.0 Hz, 2 H, Ar*H*), 6.70 (s, 2 H, Ar*H*), 5.15 (s, 4 H, C*H*_2_), 4.37 (m, 8 H, C*H*_2_).^13^C NMR (100 MHz, DMSO*-d*_6_): *δ* 153.2 (Ar*C*), 136.2 (Ar*C*), 134.9 (Ar*C*), 133.4 (Ar*C*), 130.1 (Ar*C*), 129.4 (Ar*C*), 129.2 (Ar*C*), 128.6 (Ar*C*), 128.5 (Ar*C*), 127.1 (Ar*C*), 124.7 (Ar*C*), 124.5 (Ar*C*), 122.6 (Ar*C*), 122.1 (Ar*C*), 119.3 (Ar*C*), 115.6 (Ar*C*), 67.2 (O*C*H_2_CH_2_), 52.3 (*C*H_2_), 49.0 (OCH_2_ *C*H_2_). IR (KBr, cm^−1^): 3136w, 1590w, 1271w, 1159w, 1068w, 841 s, 551 m.

### Preparation of [(*S*)-L^1^Hg(HgBr_4_)] (1)

A suspension of K_2_CO_3_ (0.179 g, 1.3 mmol), [(***S***)**-L** ^**1**^ **H**_**2**_]**·**(**PF**_**6**_)_**2**_ (0.184 g, 0.2 mmol) and HgBr_2_ (0.180 g, 0.5 mmol) in CH_3_CN/ClCH_2_CH_2_Cl (30 mL, v-v = 2:1) was stirred for 24 h at 55 °C. The resulting solution was filtered and the filtrate was concentrated to 10 mL and then Et_2_O (5 mL) was added to precipitate **1** as a pale yellow powder. Yield: 0.094 g (35%). M.p.: 162–164 °C. Anal. Calcd for C_42_H_38_Br_4_Hg_2_N_4_O_2_: C, 37.32; H, 2.83; N, 4.14%. Found: C, 37.51; H, 2.64; N, 4.33%.^1^H NMR (400 MHz, DMSO*-d*_6_): *δ* 8.06 (d, *J* = 9.2 Hz, 2 H, Ar*H*), 7.88 (d, *J* = 8.4 Hz, 2 H, Ar*H*), 7.83 (q, *J* = 8.8 Hz, 4 H, Ar*H*), 7.70 (d, *J* = 8.0 Hz, 2 H, Ar*H*), 7.58 (m, 4 H, Ar*H*), 6.89 (t, *J* = 7.2 Hz, 2 H, Ar*H*), 6.82 (t, *J* = 7.6 Hz, 2 H, Ar*H*), 6.53 (d, *J* = 8.4 Hz, 2 H, Ar*H*), 5.31 (q, *J* = 8.9 Hz, 2 H, C*H*_2_), 4.94 (m, 6 H, C*H*_2_), 4.72 (m, 2 H, C*H*_2_), 4.05 (q, *J* = 7.8 Hz, 2 H, C*H*_2_), 1.45 (t, *J* = 7.0 Hz, 6 H, C*H*_3_).^13^C NMR (100 MHz, DMSO*-d*_6_): *δ* 174.3 (2-bimi*C*), 152.3 (Ar*C*), 132.6 (Ar*C*), 132.4 (Ar*C*), 132.3 (Ar*C*), 130.3 (Ar*C*), 128.0 (Ar*C*), 126.6 (Ar*C*), 125.9 (Ar*C*), 125.8 (Ar*C*), 124.3 (Ar*C*), 123.9 (Ar*C*), 120.0 (Ar*C*), 115.8 (Ar*C*), 113.5 (Ar*C*), 113.1 (Ar*C*), 66.6 (O*C*H_2_CH_2_), 47.7 (OCH_2_ *C*H_2_), 43.9 (*C*H_2_CH_3_), 16.0 (*C*H_3_). IR (KBr, cm^−1^): 3137 m, 1598w, 1126 m, 1061 s, 951 m, 858 m, 532 m.

### Preparation of [(*S*)-L^2^Hg(HgBr_4_)] (2)

This complex was prepared in an analogous manner to that of **1**, only [(***S***)**-L** ^**2**^ **H**_**2**_]**·**(**PF**_**6**_)_**2**_ (0.164 g, 0.2 mmol) and CH_3_CN/DMSO (30 mL, v-v = 2:1) were used instead of [(***S***)**-L** ^**1**^ **H**_**2**_]**·**(**PF**_**6**_)_**2**_ and CH_3_CN/ClCH_2_CH_2_Cl. Yield: 0.107 g (43%). M.p.: 156–158 °C. Anal. Calcd for C_34_H_34_Br_4_Hg_2_N_4_O_2_: C, 32.63; H, 2.74; N, 4.48%. Found: C, 32.75; H, 2.53; N, 4.63%.^1^H NMR (400 MHz, DMSO*-d*_6_): *δ* 8.11 (d, *J* = 9.2 Hz, 2 H, Ar*H*), 7.94 (d, *J* = 8.0 Hz, 2 H, Ar*H*), 7.69 (d, *J* = 9.2 Hz, 2 H, Ar*H*), 7.42 (q, *J* = 4.8 Hz, 4 H, Ar*H*), 7.32 (t, *J* = 7.4 Hz, 2 H, Ar*H*), 7.17 (t, *J* = 7.6 Hz, 2 H, Ar*H*), 6.73 (d, *J* = 8.4 Hz, 2 H, Ar*H*), 4.97 (q, *J* = 5.6 Hz, 2 H, C*H*_2_), 4.81 (d, *J* = 11.2 Hz, 2 H, C*H*_2_), 4.39 (d, *J* = 11.2 Hz, 2 H, C*H*_2_), 4.32 (q, *J* = 7.0 Hz, 4 H, C*H*_2_), 3.95 (t, *J* = 10.8 Hz, 2 H, C*H*_2_), 1.32 (t, *J* = 4.6 Hz, 6 H, C*H*_3_).^13^C NMR (100 MHz, DMSO*-d*_6_): *δ* 174.7 (2-imi*C*), 152.0 (Ar*C*), 132.7 (Ar*C*), 129.9 (Ar*C*), 129.1 (Ar*C*), 127.9 (Ar*C*), 126.6 (Ar*C*), 124.1 (Ar*C*), 124.0 (Ar*C*), 122.9 (Ar*C*), 122.7 (Ar*C*), 119.6 (Ar*C*), 115.5 (Ar*C*), 67.4 (O*C*H_2_CH_2_), 49.4 (OCH_2_ *C*H_2_), 45.5 (*C*H_2_CH_3_), 16.7 (*C*H_3_). IR (KBr, cm^−1^): 3150 m, 1593w, 1218w, 1061 s, 950 m, 858 m, 531 m.

### Preparation of [(*S*)-L^2^Hg(HgI_4_)] (3)

A suspension of K_2_CO_3_ (0.180 g, 1.3 mmol), [(***S***)**-L** ^**2**^ **H**_**2**_]**·**(**PF**_**6**_)_**2**_ (0.164 g, 0.2 mmol) and HgI_2_ (0.221 g, 0.5 mmol) in CH_3_CN/DMSO (30 mL, v-v = 2:1) was stirred for 24 h under 60 °C. The resulting solution was filtered and the filtrate was concentrated to 10 mL and then Et_2_O (5 mL) was added to precipitate **3** as a pale yellow powder. Yield: 0.109 g (38%). M.p.: 166–168 °C. Anal. Calcd for C_34_H_34_I_4_Hg_2_N_4_O_2_: C, 28.37; H, 2.38; N, 3.89%. Found: C, 28.54; H, 2.48; N, 3.62%.^1^H NMR (400 MHz, DMSO*-d*_6_): *δ* 8.11 (d, *J* = 9.2 Hz, 2 H, Ar*H*), 7.94 (d, *J* = 8.4 Hz, 2 H, Ar*H*), 7.68 (d, *J* = 8.8 Hz, 2 H, Ar*H*), 7.41 (t, *J* = 7.4 Hz, 4 H, Ar*H*), 7.33 (t, *J* = 7.4 Hz, 2 H, Ar*H*), 7.17 (t, *J* = 7.6 Hz, 2 H, Ar*H*), 6.74 (d, *J* = 8.4 Hz, 2 H, Ar*H*), 4.95 (t, *J* = 12.6 Hz, 2 H, C*H*_2_), 4.80 (d, *J* = 12.0 Hz, 2 H, C*H*_2_), 4.38 (d, *J* = 10.8 Hz, 2 H, C*H*_2_), 4.32 (q, *J* = 7.0 Hz, 4 H, C*H*_2_), 3.95 (t, *J* = 10.8 Hz, 2 H, C*H*_2_), 1.34 (t, *J* = 7.2 Hz, 6 H, C*H*_3_).^13^C NMR (100 MHz, DMSO*-d*_6_): *δ* 175.0 (2-imi*C*), 152.5 (Ar*C*), 133.2 (Ar*C*), 130.4 (Ar*C*), 129.6 (Ar*C*), 128.4 (Ar*C*), 127.1 (Ar*C*), 124.6 (Ar*C*), 124.5 (Ar*C*), 123.4 (Ar*C*), 123.3 (Ar*C*), 120.2 (Ar*C*), 116.1 (Ar*C*), 68.0 (O*C*H_2_CH_2_), 50.0 (OCH_2_ *C*H_2_), 46.1 (*C*H_2_CH_3_), 17.1 (*C*H_3_). IR (KBr, cm^−1^): 3097 m, 1592 m, 1421 m, 1218 s, 1054 s, 819 s, 746 m, 537 m.

### Preparation of {[(*S*)-L^2^Ag](PF_6_)}_n_ (4)

Silver(I) oxide (0.167 g, 0.7 mmol) was added to an acetonitrile solution (30 mL) of [(***S***)**-L** ^**2**^ **H**_**2**_]**·**(**PF**_**6**_)_**2**_ (0.164 g, 0.2 mmol), and the suspension was stirred for 24 h under 40 °C. The resulting solution was filtered and filtrate was concentrated to 10 mL and then Et_2_O (5 mL) was added to precipitate **4** as a white powder. Yield: 0.056 g (36%). M.p.: 160–162 °C. Anal. Calcd for C_34_H_34_AgF_6_N_4_O_2_P: C, 52.12; H, 4.37; N, 7.15%. Found: C, 52.30; H, 4.58; N, 7.34%.^1^H NMR (400 MHz, DMSO*-d*_6_): *δ* 8.04 (d, *J* = 9.2 Hz, 2 H, Ph*H*), 7.92 (d, *J* = 8.4 Hz, 2 H, Ph*H*), 7.53 (d, *J* = 9.2 Hz, 2 H, Ph*H*), 7.31 (t, *J* = 7.0 Hz, 2 H, Ph*H*), 7.22 (s, 4 H, Ph*H*), 7.16 (m, 2 H, Ph*H*), 6.79 (d, *J* = 8.4 Hz, 2 H, Ph*H*), 4.59 (q, *J* = 3.7 Hz, 2 H, Ph*H*), 4.48 (m, 2 H, C*H*_2_), 4.29 (m, 2 H, C*H*_2_), 4.05 (m, 2 H, C*H*_2_), 1.30 (t, *J* = 7.4 Hz, 6 H, C*H*_3_).^13^C NMR (100 MHz, DMSO*-d*_6_): *δ* 151.7 (Ph*C*), 131.8 (Ph*C*), 128.4 (Ph*C*), 127.9 (Ph*C*), 126.7 (Ph*C*), 125.2 (Ph*C*), 123.0 (Ph*C*), 122.7 (Ph*C*), 119.9 (O*C*H_2_), 118.5 (Ph*C*), 115.0 (Ph*C*), 66.9 (O*C*H_2_CH_2_), 49.0 (OCH_2_ *C*H_2_), 44.7 (*C*H_2_CH_3_), 15.7 (*C*H_3_). IR (KBr, cm^−1^): 3169w, 1591w, 1337w, 1231w, 1153 s, 1080 s, 950 m, 841 s, 549 m.

### Preparation of [(*S*)-L^3^Ag](PF_6_) (5)

A suspension of silver oxide (0.167 g, 0.7 mmol) and precursor [(***S***)**-L** ^**3**^ **H**_**2**_]**·**(**PF**_**6**_)_**2**_ (0.189 g, 0.2 mmol) in ClCH_2_CH_2_Cl/DMSO (20 mL, v-v = 9:1) was stirred for 24 h under 40 °C. The resulting solution was filtered and filtrate was concentrated to 10 mL and then Et_2_O (5 mL) was added to precipitate **5** as a white powder. Yield: 0.058 g (32%). M.p.: 160–162 °C. Anal. Calcd for C_44_H_40_AgF_6_N_4_O_2_P: C, 58.09; H, 4.43; N, 6.15%. Found: C, 58.31; H, 4.52; N, 6.33%.^1^H NMR (400 MHz, DMSO*-d*_6_): *δ* 8.05 (d, *J* = 9.2 Hz, 2 H, Ar*H*), 7.97 (d, *J* = 8.0 Hz, 2 H, Ar*H*), 7.46 (d, *J* = 9.2 Hz, 2 H, Ar*H*), 7.35 (t, *J* = 7.4 Hz, 4 H, Ar*H*), 7.29 (m, 8 H, Ar*H*), 7.18 (t, *J* = 7.4 Hz, 5 H, Ar*H*), 6.83 (d, *J* = 8.4 Hz, 2 H, Ar*H*), 5.18 (q, *J* = 18.8 Hz, 4 H, C*H*_2_), 4.54 (m, 2 H, C*H*_2_), 4.43 (m, 2 H, C*H*_2_), 4.18 (m, 2 H, C*H*_2_), 4.01 (m, 2 H, C*H*_2_).^13^C NMR (100 MHz, DMSO*-d*_6_): *δ* 153.0 (Ar*C*), 137.1 (Ar*C*), 132.9 (Ar*C*), 130.9 (Ar*C*), 129.5 (Ar*C*), 129.1 (Ar*C*), 128.6 (Ar*C*), 127.9 (Ar*C*), 127.8 (Ar*C*), 127.3 (Ar*C*), 126.4 (Ar*C*), 124.1 (Ar*C*), 123.8 (Ar*C*), 122.0 (Ar*C*), 121.6 (Ar*C*), 119.5 (Ar*C*), 116.2 (Ar*C*), 111.1 (Ar*C*), 68.1 (O*C*H_2_CH_2_), 54.0 (*C*H_2_), 50.3 (OCH_2_ *C*H_2_). IR (KBr, cm^−1^): 3156w, 1593w, 1342w, 1153 s, 1074 s, 840 m, 550 m, 458 m.

### Fluorescence titrations

A stock solution of the host was prepared in CH_3_CN as the concentration of 1.0 × 10^−4^ mol/L. The stock solutions of the guests were prepared in CH_3_CN as the concentrations of 1.0 × 10^−3^ mol/L and 1.0 × 10^−4^ mol/L, respectively. The host solution (1.0 mL) was placed into a 10 mL volumetric flask, and the different amounts of the guest solutions (1.0 × 10^−3^ mol/L or 1.0 × 10^−4^ mol/L) were added using a microsyringe, and then diluted to 10 mL to prepare sample solutions. In the sample solutions, the concentrations of the host and the guest were 1.0 × 10^−5^ mol/L and 0–40.0 × 10^−5^ mol/L, respectively. After each addition, an equilibration time of 8–10 min was allowed before the fluorescence spectra were recorded. The fluorescence titration experiment was performed at 25 °C on a Cary Eclipse fluorescence spectrophotometer using a 1 cm path-length quartz cuvette. The sample solutions were excited at 280 nm, and the excitation and emission slits are 3 nm and 1.5 nm. The fluorescence emission spectra were recorded in the range of 300–500 nm. Statistical analysis of the data was carried out using Origin 8.0. CH_3_CN used in the titrations was freshly distilled.

### Method for Job’s plot

A stock solution of the host was prepared in CH_3_CN in the concentration of 1.0 × 10^−4^ mol/L. The stock solutions of the guest were prepared in CH_3_CN in the concentrations of 1.0 × 10^−3^ mol/L and 1.0 × 10^−4^ mol/L, respectively. In the Job’s plot experiment of **5** for H_2_PO_4_^−^, keeping the fixed overall concentration was 6.0 × 10^−5^ mol/L, and the molar fraction of H_2_PO_4_^−^ was changed from 0 to 1. In the course of preparation of sample solutions, the different amounts of host and guest solutions were placed into a 10 mL volumetric flask using a microsyringe, and then diluted to 10 mL. After each mixture, an equilibration time of 8–10 min was allowed before the absorption spectra were recorded. The absorption spectra were recorded in the range of 200–400 nm at 25 °C on a JASCO-V570 spectrometer using a 1 cm path-length quartz cuvette. Statistical analysis of the data was carried out using Origin 8.0. CH_3_CN used in the titrations was freshly distilled.

### X-ray data collection and structure determinations

X-ray single-crystal diffraction data for complexes were collected by using a Bruker Apex II CCD diffractometer at 296(2) K for [(***S***)**-L** ^**2**^ **H**_**2**_]**·**(**PF**_**6**_)_**2**_, **3** and **5**, and 173(2) K for **1**, **2** and **4** with Mo-Ka radiation (*λ* = 0.71073 Å) by *ω* scan mode. There was no evidence of crystal decay during data collection in all cases. Semiempirical absorption corrections were applied by using SADABS and the program SAINT was used for integration of the diffraction profiles^80^. All structures were solved by direct methods by using the SHELXS program of the SHELXTL package and refined with SHELXL^81^ by the full-matrix least-squares methods with anisotropic thermal parameters for all non-hydrogen atoms on *F* ^2^. Hydrogen atoms bonded to C atoms were placed geometrically and presumably solvent H atoms were first located in difference Fourier maps and then fixed in the calculated sites. Further details for crystallographic data and structural analysis are listed in Table 1 and Table 2. Figures were generated by using Crystal-Maker^82^.

**Table 1:** Summary of crystallographic data [(***S***)**-L** ^2^ **H**_2_]**·**(**PF**_6_)_2_, 1 and 2.

	[(*S*)-L^2^H_2_]·(PF_6_)_2_·4CH_2_Cl_2_	1·0.5C_2_H_4_Cl_2_	2·1.5DMSO
Chemical formula	C_136_H_144_F_48_N_16_O_8_P_8_ · 4CH_2_Cl_2_	C_42_H_38_Br_4_Hg_2_N_4_O_2_ · 0.5C_2_H_4_Cl_2_	C_34_H_34_Br_4_Hg_2_N_4_O_2_ · 1.5DMSO
Formula weight	3630.14	1401.06	1368.66
Cryst syst	Orthorhombic	Monoclinic	Monoclinic
Space group	*P*2_1_2_1_2_1_	*P*2_1_/*c*	*P*2_1_/*c*
*a*, Å	19.572(4)	21.234(1)	14.231(1)
*b*, Å	7.798(1)	25.376(2)	14.630(1)
*c*, Å	26.585(5)	17.109(1)	20.613(1)
*α*, deg	90	90	90
*β*, deg	90	111.9(1)	96.6(1)
*γ*, deg	90	90	90
*V*, Å^3^	4057.9(1)	8548.3(1)	4263.4(6)
*Z*	1	8	4
*D*_calcd_, Mg m^−3^	1.485	2.177	2.132
Abs coeff, mm^−1^	0.332	11.019	11.056
*F*(000)	1856	5272	2580
Cryst size, mm	0.15 × 0.14 × 0.13	0.18 × 0.17 × 0.15	0.15 × 0.14 × 0.13
*θ*_min_, *θ*_max_, deg	1.29, 25.00	1.03, 25.01	1.71, 25.09
*T*, K	296(2)	173(2)	173(2)
No. of data collected	20870	43821	21476
No. of unique data	7132	15032	7479
No. of refined params	626	1013	493
Goodness-of-fit on *F* ^2^ ^a^	1.015	1.017	1.017
Final *R* indices^b^ [*I* > 2*σ*(*I*)]			
*R*_1_	0.0578	0.0484	0.0482
*wR*_2_	0.1639	0.1147	0.1147
*R* indices (all data)			
*R*_1_	0.0830	0.0790	0.0685
*wR*_2_	0.1895	0.1307	0.1252

^a^ *GOF* = [Σ*w(F*_o_^2^ − *F*_c_^2^)^2^/(*n* − *p*)]^1/2^, where *n* is the number of reflection and *p* is the number of parameters refined. ^b^ *R*_1_ = Σ(*||F*_o_ *|* − *|F*_c_ *||*)/Σ*|F*_o_ *|*; *wR*_2_ = [Σ[*w*(*F*_o_^2^ − *F*_c_^2^)^2^]/ Σ*w*(*F*_o_^2^)^2^]^1/2^.

**Table 2:** Summary of crystalographic data for **3**–**5**.

	3 · DMSO	4 · CH_3_CN	5 · 0.5DMSO
Chemical formula	C_34_H_34_I_4_Hg_2_N_4_O_2_ · DMSO	C_34_H_34_AgF_6_N_4_O_2_P · CH_3_CN	C_44_H_40_AgF_6_N_4_O_2_P · 0.5DMSO
Formula weight	1517.56	824.54	946.69
Cryst syst	Monoclinic	Orthorhombic	Monoclinic
Space group	*P*2_1_/*c*	*P*2_1_2_1_2_1_	*C*2*/c*
*a*, Å	14.689(1)	11.451(7)	29.192(3)
*b*, Å	15.044(1)	14.521(9)	11.684(1)
*c*, Å	21.269(2)	21.042(1)	24.881(2)
*α*, deg	90	90	90
*β*, deg	97.7(2)	90	93.7 (2)
*γ*, deg	90	90	90
*V*, Å^3^	4657.7(9)	3499.3(4)	8468.7(1)
*Z*	4	4	8
*D*_calcd_, Mg m^−3^	2.164	1.565	1.485
Abs coeff, mm^−1^	9.315	0.695	0.609
*F*(000)	2784	1680	3864
Cryst size, mm	0.18 × 0.17 × 0.16	0.18 × 0.17 × 0.15	0.18 × 0.17 × 0.15
*θ*_min_, *θ*_max_, deg	1.66, 25.01	1.70, 25.01	1.64, 25.01
*T*, K	296(2)	173(2)	296(2)
No. of data collected	23342	18133	24112
No. of unique data	8201	6168	7450
No. of refined params	470	463	570
Goodness-of-fit on *F* ^2^ ^a^	1.069	1.043	1.031
Final *R* indices^b^ [*I* > 2*σ*(*I*)]			
*R*_1_	0.0407	0.0384	0.0523
*wR*_2_	0.1034	0.0966	0.1480
*R* indices (all data)			
*R*_1_	0.0520	0.0427	0.0672
*wR*_2_	0.1082	0.0999	0.1626

^a^ *GOF* = [Σ*w(F*_o_^2^ − *F*_c_^2^)^2^/(*n* − *p*)]^1/2^, where *n* is the number of reflection and *p* is the number of parameters refined. ^b^ *R*_1_ = Σ(*||F*_o_ *|* − *|F*_c_ *||*)/Σ*|F*_o_ *|*; *wR*_2_ = [Σ[*w*(*F*_o_^2^ − *F*_c_^2^)^2^]/ Σ*w*(*F*_o_^2^)^2^]^1/2^.

## Electronic supplementary material

Supplementary Information

## References

[1] Bianchi, A., Bowman-James, K. & Garcia-Espana, E. *Supramolecular chemistry of anions*, Wiley-VCH, New York (1997).

[2] Gupta VK, Goyal RN, Sharma RA (2008). Anion recognition using newly synthesized hydrogen bonding disubstituted phenylhydrazone-based receptors: Poly(vinylchloride)-based sensor for acetate. Talanta.

[3] Jain AK, Gupta VK, Raisoni JR (2006). Anion recognition using newly synthesized hydrogen bonding diamide receptors: PVC based sensors for carbonate. Electrochim. Acta.

[4] Jain AK, Gupta VK, Raisoni JR (2006). A newly synthesized macrocyclic dithioxamide receptor for phosphate sensing. Talanta.

[5] Rurack K, Resch-Genger U (2002). Rigidization, preorientation and electronic decoupling-the ‘magic triangle’ for the design of highly efficient fluorescent sensors and switches. Chem. Soc. Rev..

[6] Sessler JL, Seidel D (2003). Synthetic expanded porphyrin chemistry. Angew. Chem., Int. Ed..

[7] Schmuck C (2006). How to improve guanidinium cations for oxoanion binding in aqueous solution?: The design of artificial peptide receptors. Coord. Chem. Rev..

[8] Moss B (1996). A land awash with nutrients-the problem of eutrophication. Chem. Ind..

[9] Jose DA (2007). colorimetric sensor for ATP in aqueous solution. Org. Lett..

[10] Kim HN (2011). Fluorescent sensing of triphosphate nucleotides via anthracene derivatives. J. Org. Chem..

[11] Hancock RD (2013). The pyridyl group in ligand design for selective metal ion complexation and sensing. Chem. Soc. Rev..

[12] Formica M, Fusi V, Giorgi L, Micheloni M (2012). New fluorescent chemosensors for metal ions in solution. Coord. Chem. Rev..

[13] Song HB, Fan DN, Liu YQ, Zi GF (2013). Synthesis, structure, and catalytic activity of nickel complexes with new chiral binaphthyl-based NHC-ligands. J. Organomet. Chem..

[14] Marion N, Nolan SP (2008). Well-defined N-heterocyclic carbenes-Palladium(II) precatalysts for cross-coupling reactions. Acc. Chem. Res..

[15] Samojłowicz C, Bieniek M, Grela K (2009). Ruthenium-based olefin metathesis catalysts bearing N-heterocyclic carbene ligands. Chem. Rev..

[16] Liu B, Xu D, Chen WZ (2011). Facile synthesis of metal N-heterocyclic carbene complexes. Chem. Commun..

[17] Zhao DW, Xie YF, Song HB, Tang LF (2012). Synthesis and reactivity of bis(3,5-dimethylpyrazol-1-yl)methanes functionalized by 2-halophenyl groups on the methine carbon. J. Organomet. Chem..

[18] Zinner SC, Rentzsch CF, Herdtweck E, Herrmann WA, Kuehn FE (2009). N-Heterocyclic carbenesofiridium(I): Ligand effects on the catalytic activity in transfer hydrogenation. Dalton Trans..

[19] Zhang XQ, Qiu YP, Rao B, Luo MM (2009). Organometallics.

[20] Mahapatra AK, Hazra G, Sahoo P (2012). First theophylline-based ratiometric fluorescent synthetic receptor for selective recognition of dihydrogen phosphate and biological phosphate ions. Bioorg. Med. Chem. Lett..

[21] Gil-Ramírez G, Escudero-Adán EC, Benet-Buchholz J, Ballester P (2008). Quantitative evaluation of anion-π interactions in solution. Angew. Chem., Int. Ed..

[22] Guha S, Saha S (2010). Fluoride ion sensing by an anion-π interaction. J. Am. Chem. Soc..

[23] Nair AK, Neelakandan PP, Ramaiah D (2009). A supramolecular Cu(II) metallocyclophane probe for guanosine 5′-monophosphate. Chem. Commun..

[24] Sakamato T, Ojida A, Hamachi I (2009). Molecular recognition, fluorescence sensing, and biological assay of phosphate anion derivatives using artificial Zn(II)-dpa complexes. Chem. Commun..

[25] Natale D, Mareque-Rivas JC (2008). The combination of transition metal ions and hydrogen-bonding interactions. Chem. Commun..

[26] Xu Z (2009). An NBD-based colorimetric and fluorescent chemosensor for Zn^2+^ and its use for detection of intracellular zinc ions. Tetrahedron.

[27] Kim KM, Oh DJ, Ahn KH (2011). Competition assay of thymidine phosphates with a (Zn^2+^-cyclen)-lumazine ensemble. Bull. Korean Chem. Soc..

[28] Tang LJ, Zhang H, Guo Z, Qian J (2009). A new chemo-sensing ensemble for fluorescent recognition of pyrophosphate in water at physiological pH. Tetrahedron Lett..

[29] Aoki S (2005). A luminescence sensor of inositol 1,4,5-triphosphate and its model compound by ruthenium-templated assembly of a bis(Zn^2+^-cyclen) complex having a 2,2′-bipyridyl linker (cyclen = 1,4,7,10-tetraazacyclododecane). J. Am. Chem. Soc..

[30] Kitamura M, Nishimoto H, Aoki K, Tsukamoto M, Akoi S (2010). Molecular recognition of inositol 1,4,5-trisphosphate and model compounds in aqueous solution by ditopic Zn^2+^ complexes containing chiral linkers. Inorg. Chem..

[31] Aoki S, Kimura E (2002). Recent progress in artificial receptors for phosphate anions in aqueous solution. J. Biotechnol..

[32] Liu QX (2015). Structures of NHC Hg(II) and Ag(I) complexes and selective recognition of nitrate anion. CrystEngComm.

[33] Liu QX, Wei Q, Liu R, Zhao XJ, Zhao ZX (2015). NHC macrometallocycles of mercury(II) and silver(I): synthesis, structural studies and recognition of Hg(II) complex 4 for silver ion. RSC Adv..

[34] Lin CX (2013). Dinuclear Ag(I) metallamacrocycles of bis-N-heterocyclic carbenes bridged by calixarene fragments: synthesis, structure and chemosensing behavior. CrystEngComm.

[35] Arnold PL, Casely IJ (2009). F-Block N-heterocyclic carbene complexes. Chem. Rev..

[36] Liu XL, Chen WZ (2012). Pyridazine-based N-heterocyclic carbene complexes and ruthenium-catalyzed oxidation reaction of alkenes. Organometallics.

[37] Han YF, Jin GX, Hahn FE (2013). Postsynthetic modification of dicarbene-derived metallacycles via photochemical [2 + 2] cycloaddition. J. Am. Chem. Soc..

[38] Chen JH, Zhang XQ, Feng Q, Luo MM (2006). Novel hexadentate imidazolium salts in the rhodium-catalyzed addition of arylboronic acids to aldehydes. J. Organomet. Chem..

[39] Wang JW, Meng FH, Zhang LF (2009). Suzuki coupling reaction of aryl halides catalyzed by an N-heterocyclic carbene-PdCl_2_ species based on a porphyrin at room temperature. Organometallics.

[40] Song HB, Liu YQ, Fan DN, Zi GF (2011). Synthesis, structure, and catalytic activity of rhodium complexes with new chiral binaphthyl-based NHC-ligands. J. Organomet. Chem..

[41] Han YF, Jin GX, Daniliuc CG, Hahn FE (2015). Reversible photochemical modifications in dicarbene-derived metallacycles with coumarin pendants. Angew. Chem., Int. Ed..

[42] Van Veldhuizen JJ, Campbell JE, Giudici RE, Hoveyda AH (2005). A readily available chiral Ag-based N-heterocyclic carbene complex for use in efficient and highly enantioselective Ru-catalyzed olefin metathesis and Cu-catalyzed allylic alkylation reactions. J. Am. Chem. Soc..

[43] Lin JCY (2009). Coinage metal-N-heterocyclic carbene complexes. Chem. Rev..

[44] Crudden CM, Allen DP (2004). Stability and reactivity of N-heterocyclic carbene complexes. Coord. Chem. Rev..

[45] Jiang YS, Chen WZ, Lu WM (2013). Synthesis of 3-arylcoumarins through N-heterocyclic carbene catalyzed condensation and annulation of 2-chloro-2-arylacetaldehydes with salicylaldehydes. Tetrahedron.

[46] Wang JW, Li QS, Xu FB, Song HB, Zhang ZZ (2006). Synthetic and structural studies of silver(I)- and gold(I)-containing N-heterocyclic carbene metallacrown ethers. Eur. J. Org. Chem..

[47] Wang X, Liu S, Weng LH, Jin GX (2006). A trinuclear silver(I) functionalized N-heterocyclic carbene complex and its use in transmetalation: structure and catalytic activity for olefin polymerization. Organometallics.

[48] Gade LH, Bellemin-Laponnaz S (2007). Mixed oxazoline-carbenes as stereodirecting ligands for asymmetric catalysis. Coord. Chem. Rev..

[49] Liu B, Chen CY, Zhang YJ, Liu XL, Chen WZ (2013). Dinuclear copper(I) complexes of phenanthrolinyl-functionalized NHC ligands. Organometallics.

[50] Garrisen JC (2001). Synthesis and structural characterization of an imidazolium-linked cyclophane and the silver complex of an N-heterocyclic carbene-linked cyclophane. Organometallics.

[51] Tulloch AAD, Winston S, Danopoulos AA, Eastham G, Hursthouse MB (2003). Functionalised and chelate heterocyclic carbene complexes of palladium; synthesis and structural studies. Dalton Trans..

[52] Guerret O (1997). 1,2,4-Triazole-3,5-diylidene: A building block for organometallic polymer synthesis. J. Am. Chem. Soc..

[53] Lee CK, Lee KM, Lin IJB (2002). Inorganic-organic hybrid lamella of di- and tetranuclear silver-carbene complexes. Organometallics.

[54] Arduengo III AJ, Dias HVR, Calabrese JC, Davidson F (1993). Homoleptic carbene-silver(I) and carbene-copper(I) complexes. Organometallics.

[55] Ku RZ (1999). Metal ion mediated transfer and cleavage of diaminocarbene ligands. Organometallics.

[56] Pickering AL, Seeber G, Long DL, Cronin L (2005). The importance of π-π, π-CH and N-CH interactions in the crystal packing of Schiff-base derivatives of cis,cis- and cis,trans-1,3,5-triaminocyclohexane. CrystEngComm.

[57] Mohomed K, Gerasimov TG, Abourahma H, Zaworotko MJ, Harmon JP (2005). Nanostructure matrix interactions in methacrylate composites. Mat. Sci. Eng. A..

[58] Liu QX (2011). Mercury(II), copper(II) and silver(I) complexes with ether or diether functionalized bis-NHC ligands: synthesis and structural studies. CrystEngComm.

[59] Schonherr HJ, Wanzlick HW (1970). Chemistry of nucleophilic carbenes. XX. HX-Elimination from 1,3-diphenylimidazolium salts. Mercurysalt-carbene complexes. Chem. Ber..

[60] Luger P, Ruban G (1971). Crystal structure of a mercury salt-carbene complex. Acta Crystallogr., Sect. B..

[61] Lee KM, Chen JCC, Lin IJB (2001). Helical mono and dinuclear mercury(II) N-heterocycliccarbene complexes. J. Organomet. Chem..

[62] Lin J, Dong GY (2007). {1,1′-Bis(1-naphthylmethyl)-3,3′-[1,1′-binaphthyl-2,2′-diyldi(oxyethylene)]di-1H-imidazol-2-yl}mercury(II) bis(hexafluoridophosphate)acetonitrile 3.5-solvate. Acta Crystallogr., Sect..

[63] Mahmoudi G, Morsali A (2007). Counter-ion influence on the coordination mode of the 2,5-bis(4-pyridyl)-1,3,4-oxadiazole (bpo) ligand in mercury(II) coordination polymers, [Hg(bpo)_n_X_2_]: X = I^−^, Br^−^, SCN^−^, N^3−^ and NO^2−^; spectroscopic, thermal, fluorescence and structural studies. CrystEngComm.

[64] Liu QX (2011). N-heterocyclic carbene copper(I), mercury(II) and silver(I) complexes containing durene linker: Synthesis and structural studies. CrystEngComm.

[65] Liu QX, Yin LN, Feng JC (2007). New N-heterocyclic carbene silver(I) and mercury(II) 2-D supramolecular layers by the π-π stacking interactions. J. Organomet. Chem..

[66] Grdenić D (1981). Connections in the crystal structures of mercury compounds. Structural studies of molecules of biological interest.

[67] Santra R, Banerjee K, Biradha K (2011). Weak Ag···Ag and Ag···π interactions in templating regioselective single and double [2 + 2] reactions of N,N’-bis(3-(4-pyridyl)acryloyl)-hydrazine:synthesis of an unprecedented tricyclohexadecane ring system. Chem. Commun..

[68] Habata Y (2012). Argentivorous molecules: structural evidence for Ag^+^-π interactions in solution. Org. Lett..

[69] Nielsen DJ, Cavell KJ, Skelton BW, White AH (2006). Silver(I) and palladium(II) complexes of an ether-functionalized quasi-pincer bis-carbene ligand and its alkyl analogue. Organometallics.

[70] Venkatasetty HV, Saathoff DJ (1978). The conductance, cyclic voltammetric, and infrared spectral studies of electrolytes in dimethyl sulfoxide. J. Electrochem. Soc..

[71] Arunkumar E, Ajayaghosh A, Daub J (2005). Selective calcium ion sensing with a bichromophoric squaraine foldamer. J. Am. Chem. Soc..

[72] Madhu S, Ravikanth M (2014). Boron-dipyrromethene based reversible and reusable selective chemosensor for fluoride detection. Inorg. Chem..

[73] Velmurugan K, Mathankumar S, Santoshkumar S, Amudha S, Nandhakumar R (2015). Specific fluorescent sensing of aluminium using naphthalene benzimidazole derivative in aqueous media. Spectrochimica Acta A.

[74] Connors, K. A. Binding constants, the measurement of molecular complex stability, John Wiley & Sons: New York, (1987).

[75] Valeur, B. In *Molecular fluorescence principles & applications*, Wiley-VCH Verlag GmbH: New York (2001).

[76] Caballero A (2005). Highly selective chromogenic and redox or fluorescent sensors of Hg^2+^ in aqueous environment based on 1,4-disubstituted azines. J. Am. Chem. Soc..

[77] Polster, J. & Lachmann, H. *Spectrometric titrations*, VCH: Weinheim, Germany (1989).

[78] Wang J, Bodige SG, Watson WH, Gutsche CD (2000). Complexation of fullerenes with 5,5′-biscalix[5]arene. J. Org. Chem..

[79] Shyamaprosad G, Anita H, Rinku C, Hoong KF (2009). Recognition of carboxylate anions and carboxylic acids by selenium-based new chromogenic fluorescent sensor: A remarkable fluorescence enhancement of hindered carboxylates. Org. Lett..

[80] Bruker Instrumentation *SAINT Software Reference Manual*, Bruker AXS, Madison (1998).

[81] Sheldrick, G. M. SHELXTL NT (Version 5.1). Program for Solution & Refinement of Crystal Structures, University of Göttingen, Germany (1997).

[82] Palmer, D. C. *CrystalMaker 7.1.5*. *CrystalMaker Software*, Yarnton, UK (2006).

