# Supplementary material for: Synthesis and structural studies of N-heterocyclic carbene Ag(I) and Hg(II) complexes and recognition of dihydrogen phosphate anion
Source: Sci Rep. 2017 Aug 8;7:7534. doi: 10.1038/s41598-017-07961-8 (PMC5548890; doi:10.1038/s41598-017-07961-8)
Supplement: Supplementary file 1 — Supplementary Information [file 41598_2017_7961_MOESM1_ESM.doc]

**SUPPLEMENTARY INFORMATION**

**Synthesis and structural studies of** [**NHC Ag(I) and Hg(II) complexes and recognition of**](https://scifinder.cas.org/scifinder/references/answers/80EA19BCX86F35093X39FF1437426CB1336A:80EB7054X86F35093X1009661A244BE7C1B5/3.html?nav=eNpVkLEvQ0Ecx39eIyI6lEVEKgaDkNx7pa02ldBSNF5eRRGxyGkv9Xjv3XN3rXYRBgwWg7IYDDZ24k-QGIVFInZWicm9loibLvl97nPf3_f6HZoFNGEBfTEtnRrRIuHlWHRqOKLFh5dDmhaPRkPJoXA4lR6ZCKUiEl3jDNo3cBkjCztFlHEEKRLW8XZx-bl3GFOgKQPNZWyVSIVB4I8zSvYaYQfXtWDbyeuRAlBxAcAnhesCupOLCzPZ-dWMsZQ2FuTFyK5Oz2cX5zLGtIBW03YpE9LAt2AHfPIdCFAY_Z8kRalFsPPQy3Yfz78-ZJKV3ySux3Mu-UHKiiiPOaI8jxnihJUJQwVqY9NBeWrb1EE5-VnOJfnR46uL4NnrvQKKDn67mmUF08HWLKkK6NelSJUitS5SGyK1IVIbIlWSCR1a7Kpn5AK6dC-tWhKmpeqms0kKM5iv54hIVFxXhuusL-ON0b_xs_W0UnsZ6PFa-125Tv3M7yb3a6e3N2Gf1-q2X9YTGBuH-ql8A7n2nak&key=caplus_2013:911297&title=U3ludGhlc2lzIGFuZCBzdHJ1Y3R1cmFsIGNoYXJhY3Rlcml6YXRpb24gb2YgTi1oZXRlcm9jeWNsaWMgY2FyYmVuZSBzaWx2ZXIgY29tcGxleGVzIGRlcml2ZWQgZnJvbSBOLWZlcnJvY2VueWxtZXRoeWwtTictKHB5cmlkeWxtZXRoeWwpaW1pZGF6b2xpdW0gaW9kaWRlcw&launchSrc=reflist&p=1) **dihydrogen phosphate anion**

Qingxiang Liu*, Xiaoqiang Zhao, Zeliang Hu, Zhixiang Zhao, and Hong Wang

*Key Laboratory of Inorganic-Organic Hybrid Functional Materials Chemistry (Tianjin Normal University), Ministry of Education; Tianjin Key Laboratory of Structure and Performance for Functional Molecules; College of Chemistry, Tianjin Normal University, Tianjin 300387, China.*

** Corresponding author, E-mail:* [*tjnulqx@163.com*](mailto:tjnulqx@163.com)

**List of the contents**

1. CCDC numbers for **[(*S*)-L^2^H_2_]·(PF_6_)_2_** and complexes **1**-**5**

2. The data of dihedral angles and intra-molecular π-π interactions for **[(*S*)-L^2^H_2_]·(PF_6_)_2_** and complexes **1**-**5** (Table S1-Table S2)

3. The simulated and the experimental PXRD patterns for complexes **1**-**5** (Figure S1-Figure S5)

4. The curves of thermogravimetric analysis for complexes **1**-**5** (Figure S6-Figure S10)

5. The infrared spectra of **[(*S*)-L^1^H_2_]·(PF_6_)_2_**-**[(*S*)-L^3^H_2_]·(PF_6_)_2_** and complexes **1**-**5** (Figure S11-Figure S15)

6. The Figures of fluorescence and UV/vis spectroscopies for complex **5** (Figure S16-Figure S18)

7. HRMS spectrum of **5**·H_2_PO_4_^-^ (Figure S19)

8. The Figures of ^1^H NMR and ^13^C NMR spectra for precursors **[(*S*)-L^1^H_2_]·(PF_6_)_2_**~**[(*S*)-L^3^H_2_]·(PF_6_)_2_**, complexes **1**-**5** (Figure S20-Figure S35)

1. **CCDC numbers for [(*S*)-L^2^H_2_]·(PF_6_)_2_ and complexes 1-5**

CCDC 1023646, 1023636-1023638, 1023634 and 1023635 contains the supplementary crystallographic data for precursor **[(*S*)-L^2^H_2_]·(PF_6_)_2_** and complexes **1-5**. These data can be obtained free of charge via http//www.ccdc.cam.ac.uk/conts/retrieving.html, or from the Cambridge Crystallographic Data Centre, 12 Union Road, Cambridge, CB2 1EZ, UK; fax: (+44) 1223-336-033; or e-mail: deposit@ccdc.cam.ac.uk.

**2. The data of dihedral angles and intra-molecular π-π interactions for [(*S*)-L^2^H_2_]·(PF_6_)_2_ and complexes 1-5**

**Table S1.** In the same ligand of **[(*S*)-L^2^H_2_]·(PF_6_)_2_** and **1**-**5**, the dihedral angles (˚) between two [naphthalene](app:ds:naphthalene) rings (A), the dihedral angles (˚) between two benzimidazole (or imidazole) rings (B).

| Compounds | A | B |
| --- | --- | --- |
| **[(*S*)-L^2^H_2_]·(PF_6_)_2_** | 74.4(4) | 50.2(2) |
| **1** | 79.5(2) | 38.1(7) |
| **2** | 79.9(5) | 33.2(4) |
| **3** | 82.5(6) | 31.1(1) |
| **4** | 77.6(7) | 64.2(3) |
| **5** | 81.6(3) | 40.3(4) |

**Table S2.** Distances (Å) of intra-molecular π-π interactions for **[(*S*)-L^2^H_2_]·(PF_6_)_2_** and complexes **1**-**3**.

| Compounds | π-π | |
| --- | --- | --- |
|  | Face-to-face | Center-to-center |
| **[(*S*)-L^2^H_2_]·(PF_6_)_2_** | 3.535(6) (imidazole and naphalene) | 3.859(5) (imidazole and naphalene) |
| **1** | 3.560(1) (benzimidazole and naphalene) | 3.931(3) (benzimidazole and naphalene) |
| **2** | 3.420(1) (imidazole and naphalene) | 3.799(2) (imidazole and naphalene) |
| **3** | 3.537(1) (imidazole and naphalene) | 3.858(2) (imidazole and naphalene) |

**3. The simulated and the experimental PXRD patterns for complexes 1-5**


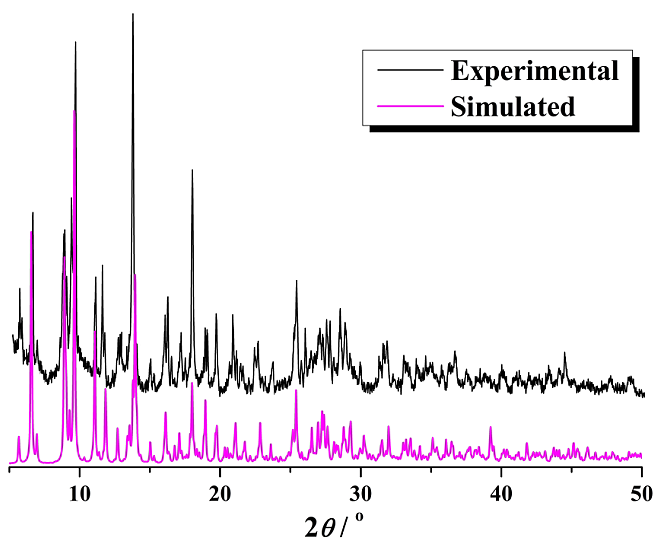


**Figure S1.** The simulated (purple) and the experimental (black) PXRD patterns of **1**.


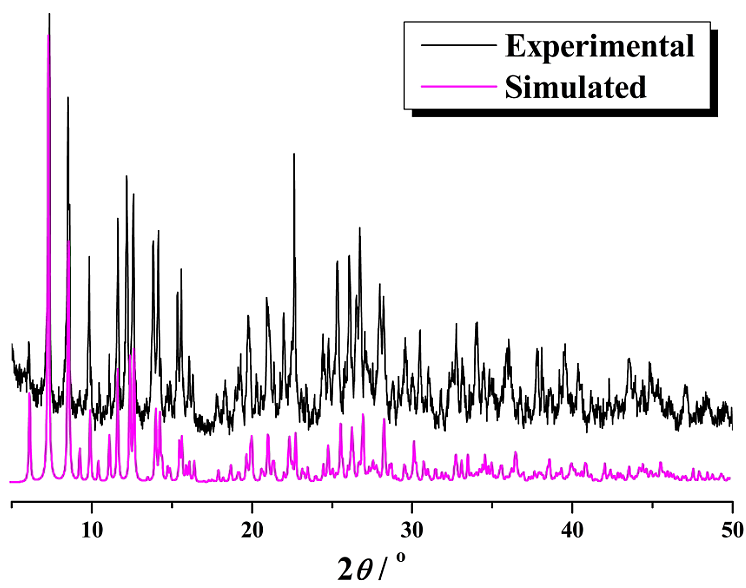


**Figure S2.** The simulated (purple) and the experimental (black) PXRD patterns of **2**.


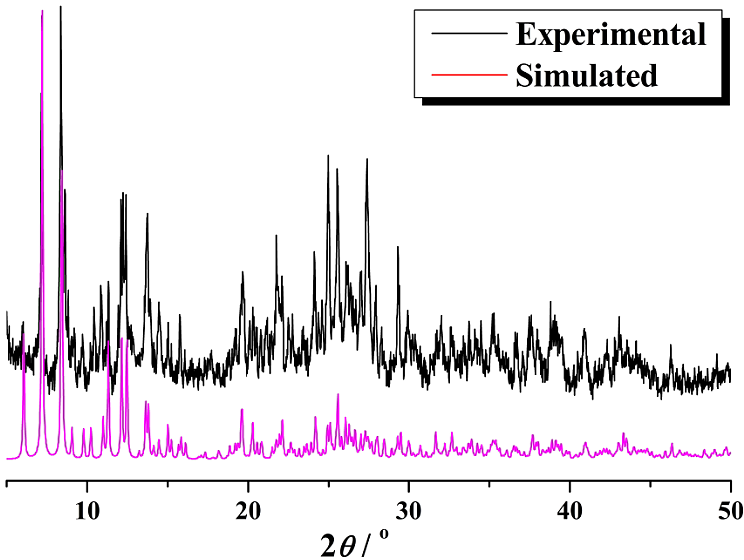


**Figure S3.** The simulated (purple) and the experimental (black) PXRD patterns of **3**.


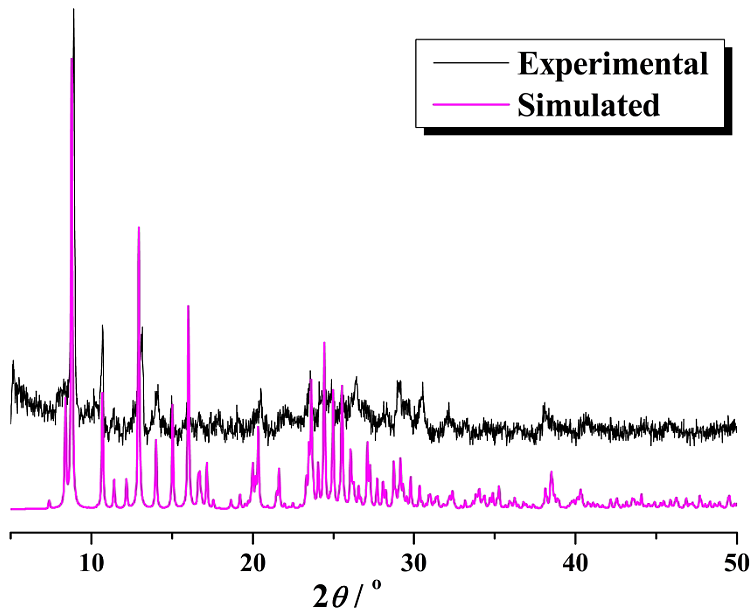


**Figure S4.** The simulated (purple) and the experimental (black) PXRD patterns of **4**.


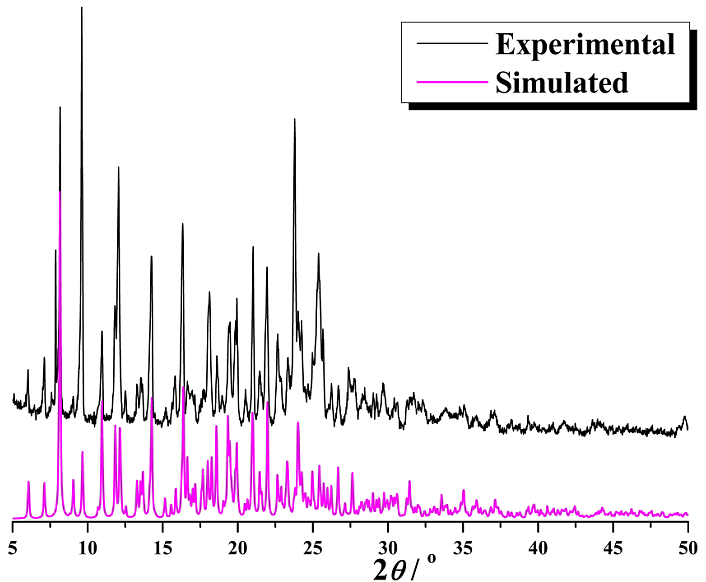


**Figure S5.** The simulated (purple) and the experimental (black) PXRD patterns of **5**.

**4. The curves of thermogravimetric analysis for complexes 1-5**


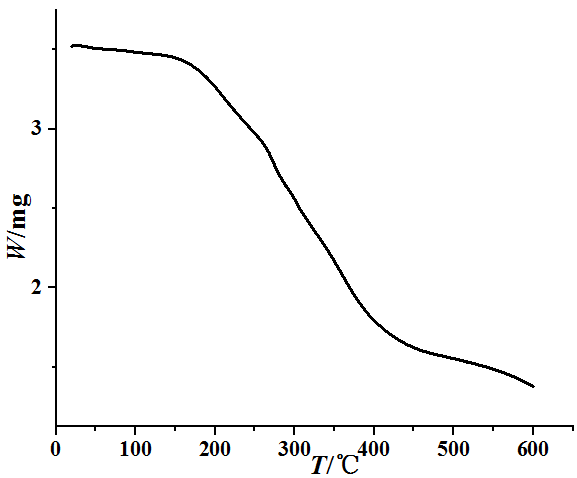


**Figure S6.** TG curves from room temperature to 600 ˚C for **1**.


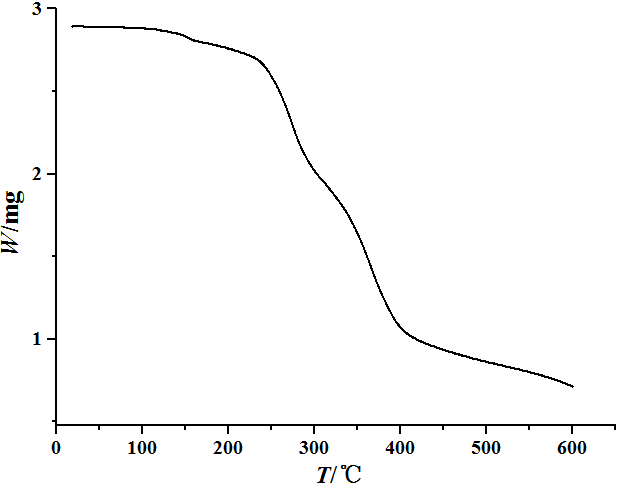


**Figure S7.** TG curves from room temperature to 600 ˚C for **2**.


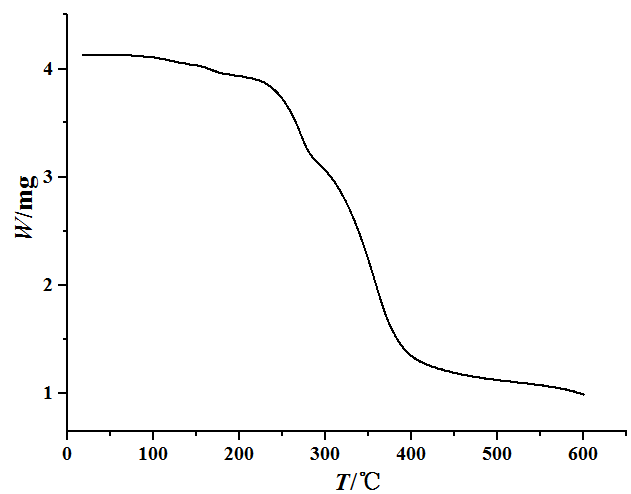


**Figure S8.** TG curves from room temperature to 600 ˚C for **3**.


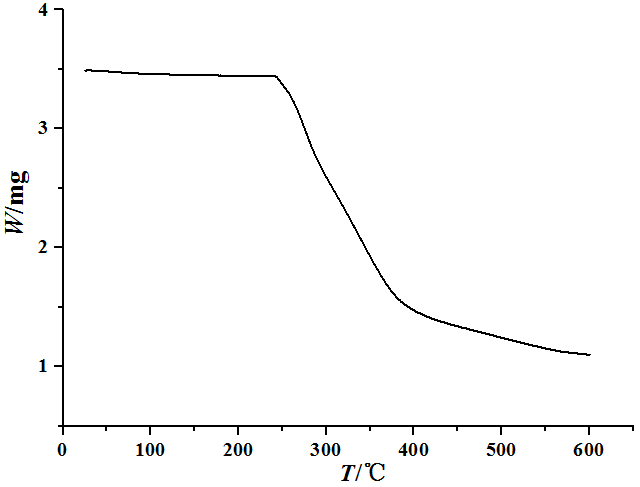


**Figure S9.** TG curves from room temperature to 600 ˚C for **4**.


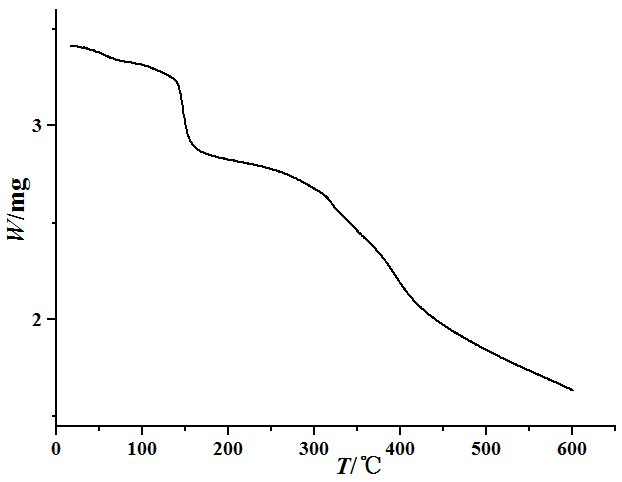


**Figure S10.** TG curves from room temperature to 600 ˚C for **5**.

**5. The infrared spectra of [(*S*)-L^1^H_2_]·(PF_6_)_2_-[(*S*)-L^3^H_2_]·(PF_6_)_2_ and complexes 1-5**


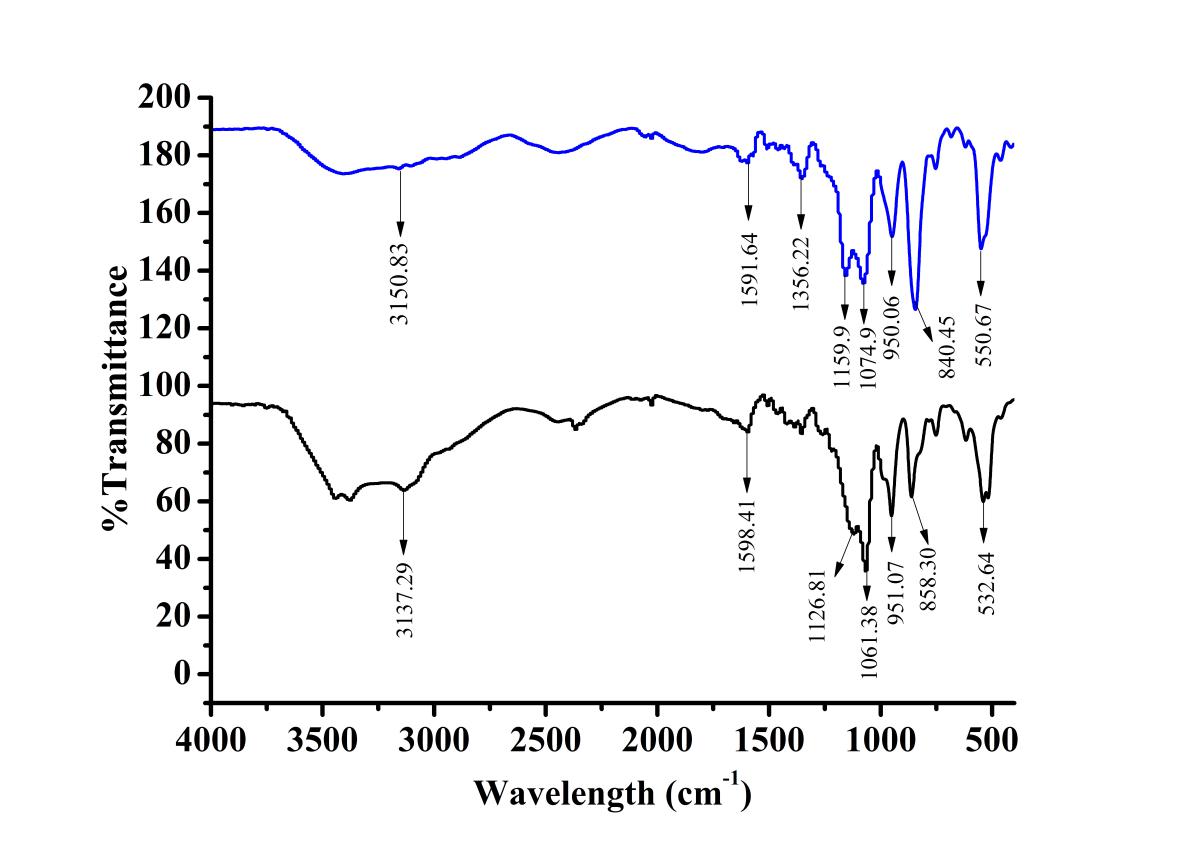


**Figure S11.** Infrared spectroscopy of **[(S)-L^1^H_2_]·(PF_6_)_2_** (top) and complex **1** (bottom).


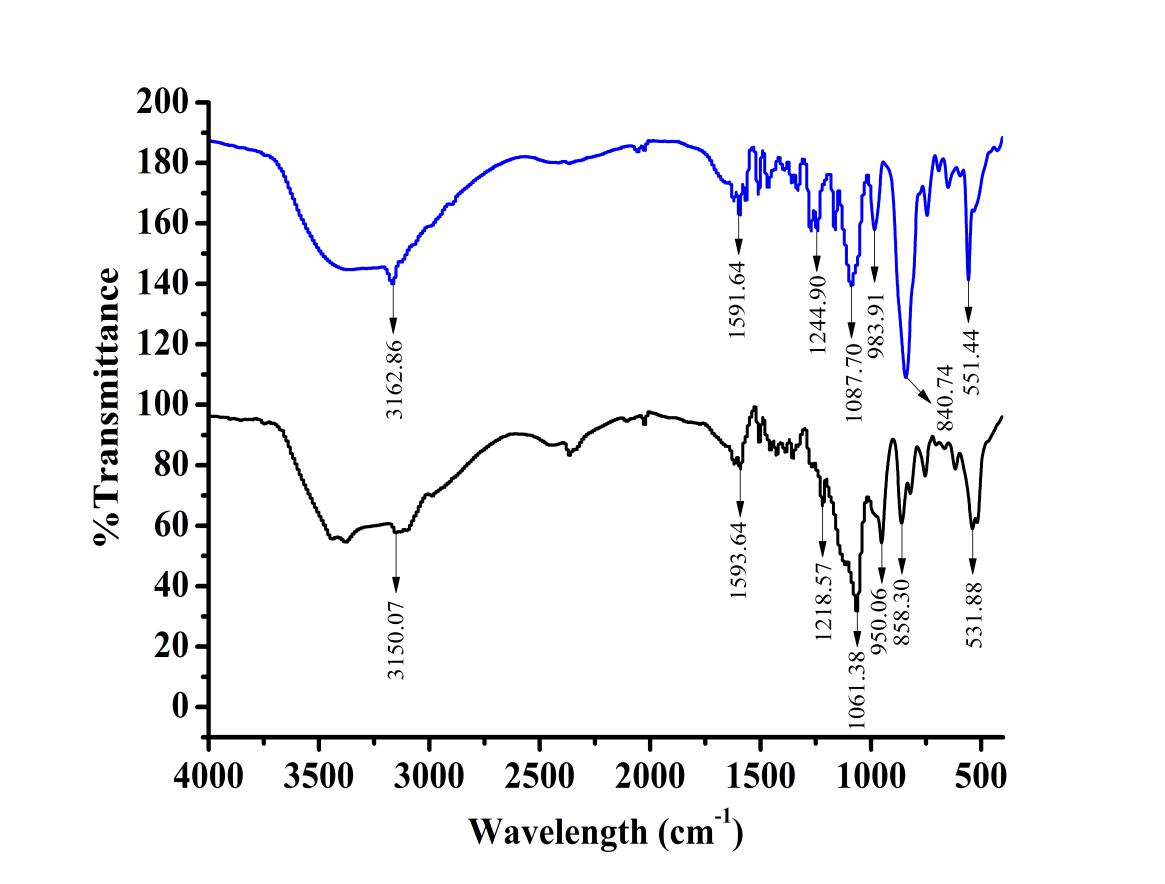


**Figure S12.** Infrared spectroscopy of **[(S)-L^2^H_2_]·(PF_6_)_2_** (top) and complex **2** (bottom).


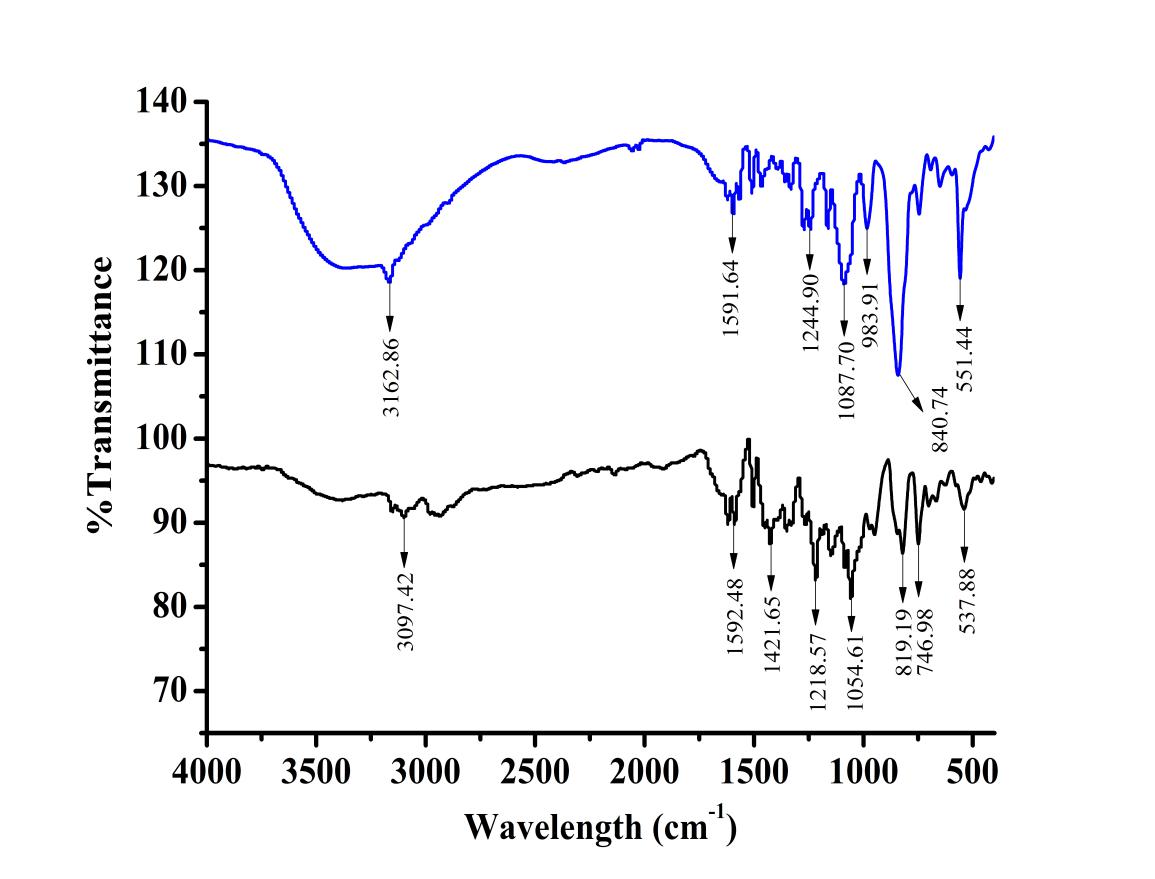


**Figure S13.** Infrared spectroscopy of **[(S)-L^2^H_2_]·(PF_6_)_2_** (top) and complex **3** (bottom).


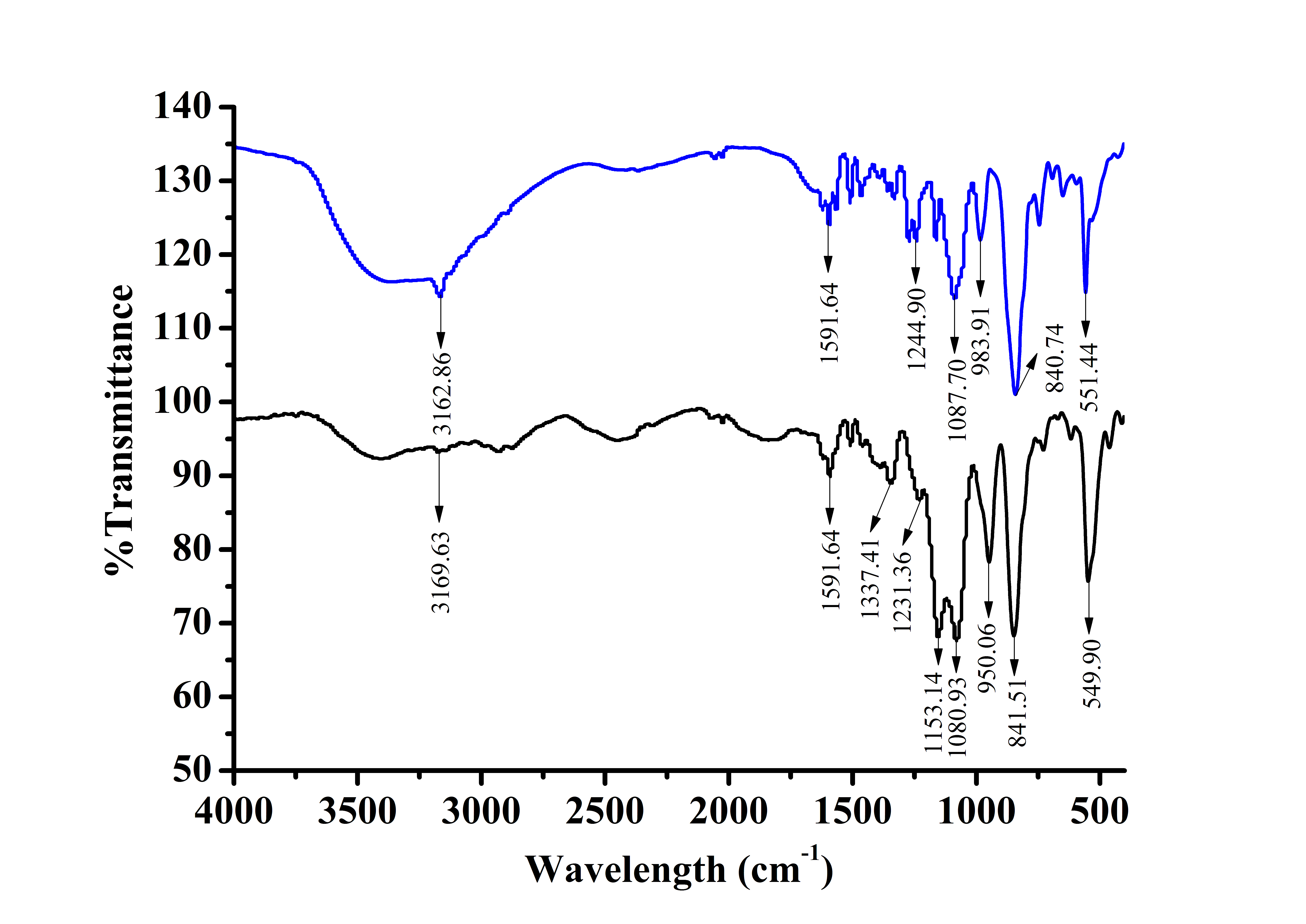


**Figure S14.** Infrared spectroscopy of **[(S)-L^2^H_2_]·(PF_6_)_2_** (top) and complex **4** (bottom).


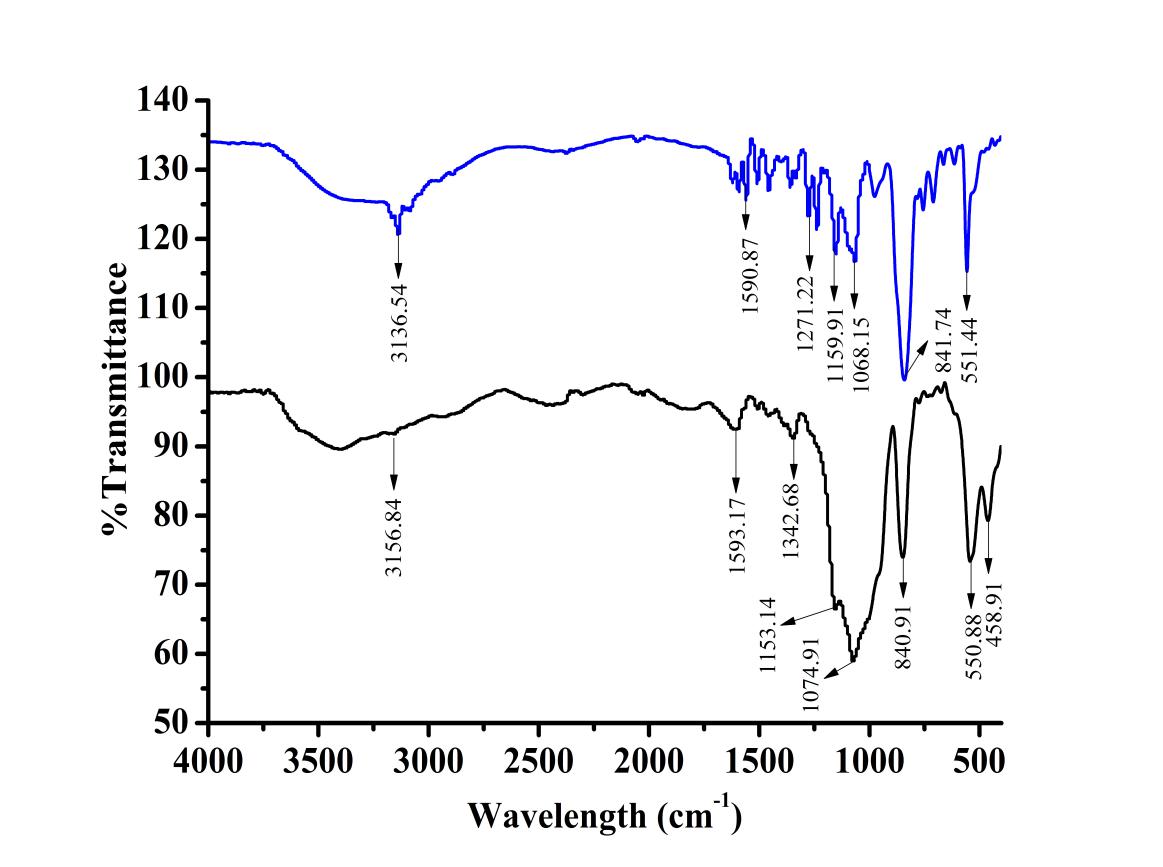


**Figure S15.** Infrared spectroscopy of **[(S)-L^3^H_2_]·(PF_6_)_2_** (top) and complex **5** (bottom).

**6. The figures of fluorescence and UV/vis spectroscopies for complex 5**


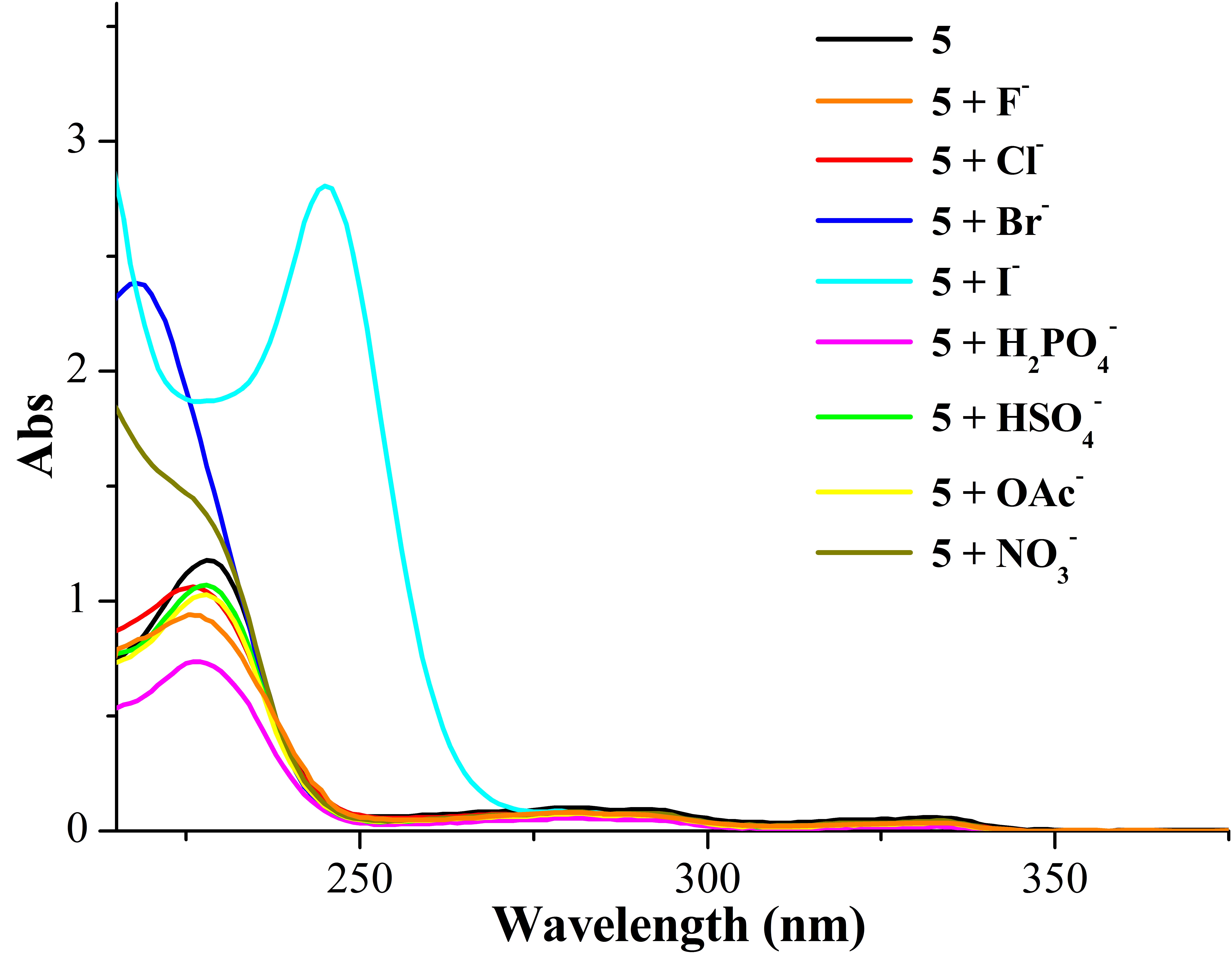


**Figure S16.** UV/vis absorption spectra of **5** (1 × 10^-5^ mol/L) and upon the addition of salts (20 equiv.) of F^-^, Cl^-^, Br^-^, I^-^, H_2_PO_4_^-^, HSO_4_^-^, OAc^-^ and NO_3_^-^ in CH_3_CN at 25 ˚C.


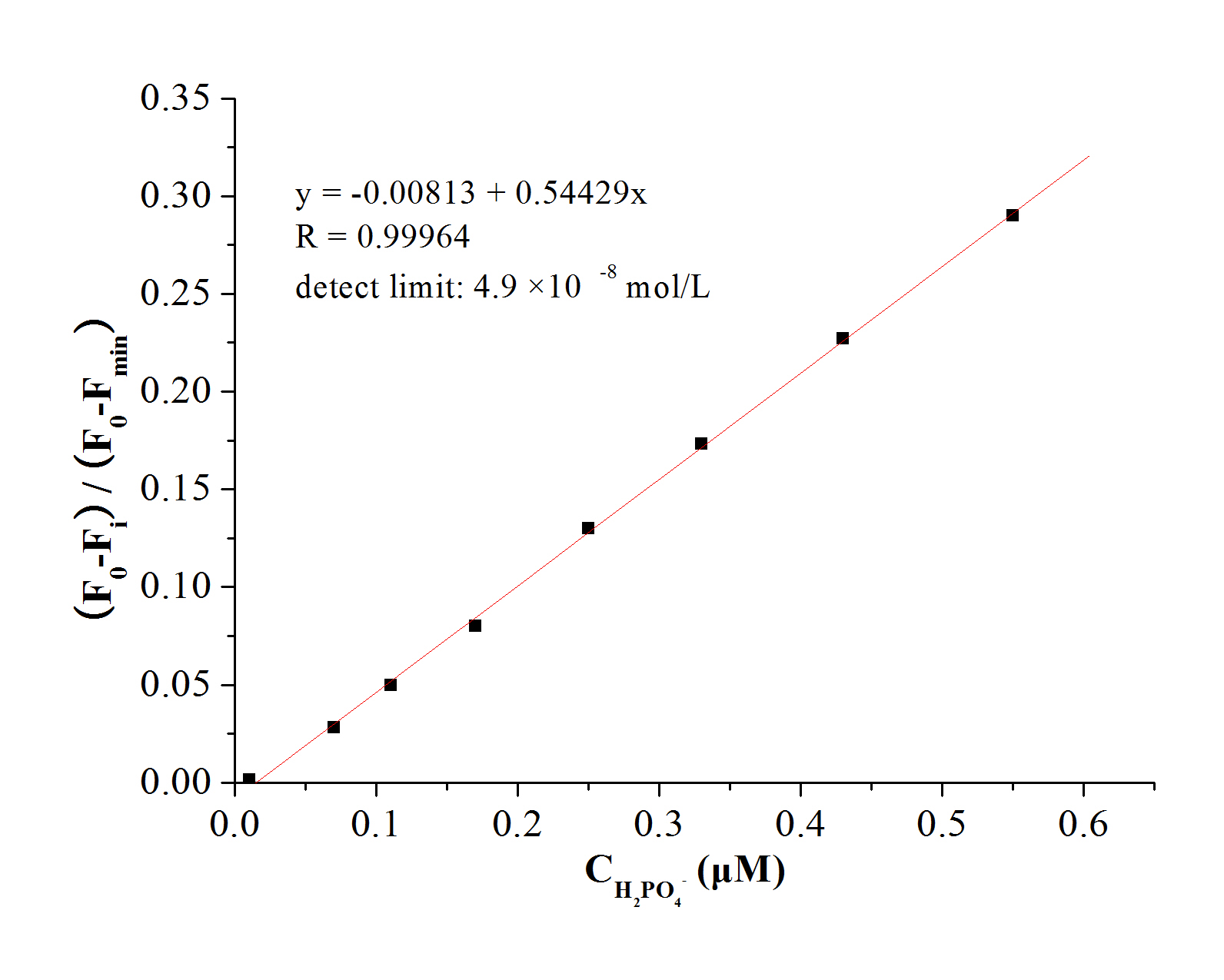


**Figure S17.** Emission (at 360 nm) of **5** at different concentrations of H_2_PO_4_^-^ (0, 0.07, 0.11, 0.17, 0.25, 0.33, 0.43, 0.55 × 10^-5^ mol/L) added, normalized between the minimum emission (0.0 μM) and the emission (0.55 × 10^-5^ mol/L). The detection limit was determined to be 4.9 × 10^-8^ mol/L.


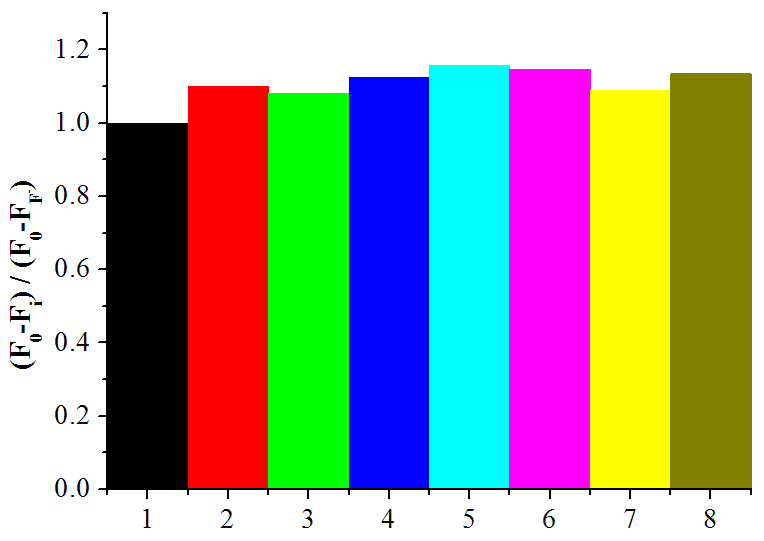


**Figure S18.** Change ratio (F_0_-F_i_)/(F_0_-F_F_^-^) of fluorescence intensity of **5** upon addition of 10 equiv. H_2_PO_4_^-^ in the presence of 10 equiv. of background anions. 1: H_2_PO_4_^-^; 2: H_2_PO_4_^-^ + Cl^-^; 3: H_2_PO_4_^-^ + Br^-^; 4: H_2_PO_4_^-^ + I^-^; 5: H_2_PO_4_^-^ + F^-^; 6: H_2_PO_4_^-^ + HSO_4_^-^; 7: H_2_PO_4_^-^ + OAc^-^, 8: H_2_PO_4_^-^ + NO_3_^-^ in CH_3_CN at 25 ˚C.

**7. HRMS spectrum of 5·H_2_PO_4_^-^**


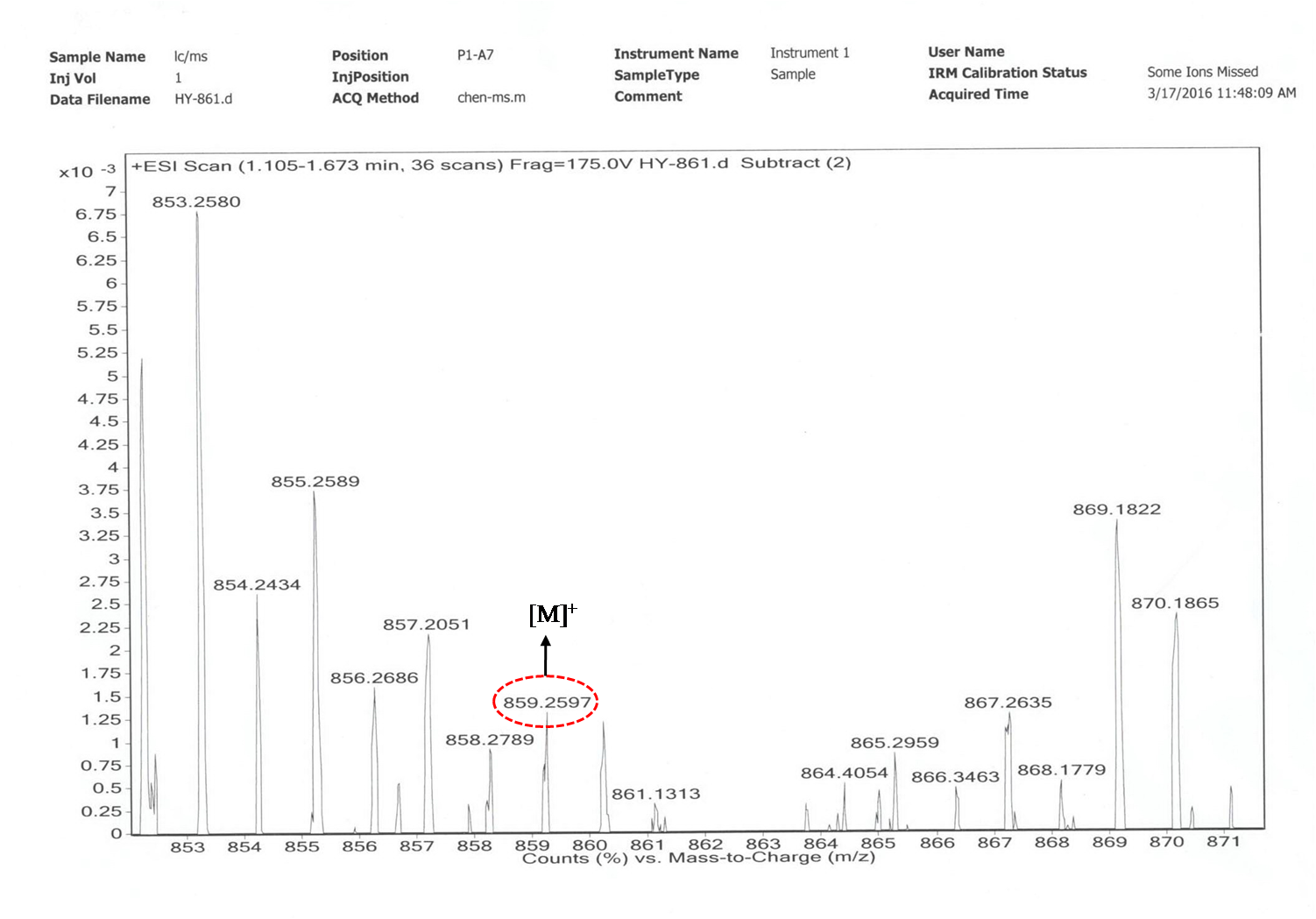


**Figure S19.** HRMS spectrum of **5**·H_2_PO_4_^-^.

**8. The figures of ^1^H NMR and ^13^C NMR spectra for precursors [(*S*)-L^1^H_2_]·(PF_6_)_2_~[(*S*)-L^3^H_2_]·(PF_6_)_2_ and complexes 1-5**.

**
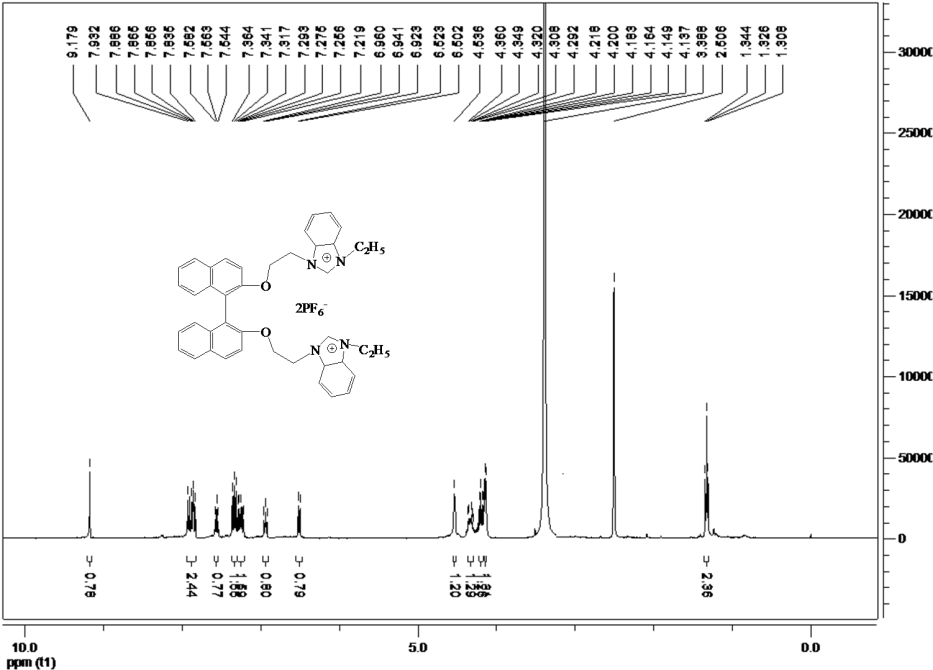
**

**Figure S20.** The ^1^H NMR (400 MHz, DMSO-*d*_6_) spectrum of **[(*S*)-L^1^H_2_]·(PF_6_)_2_**.

**
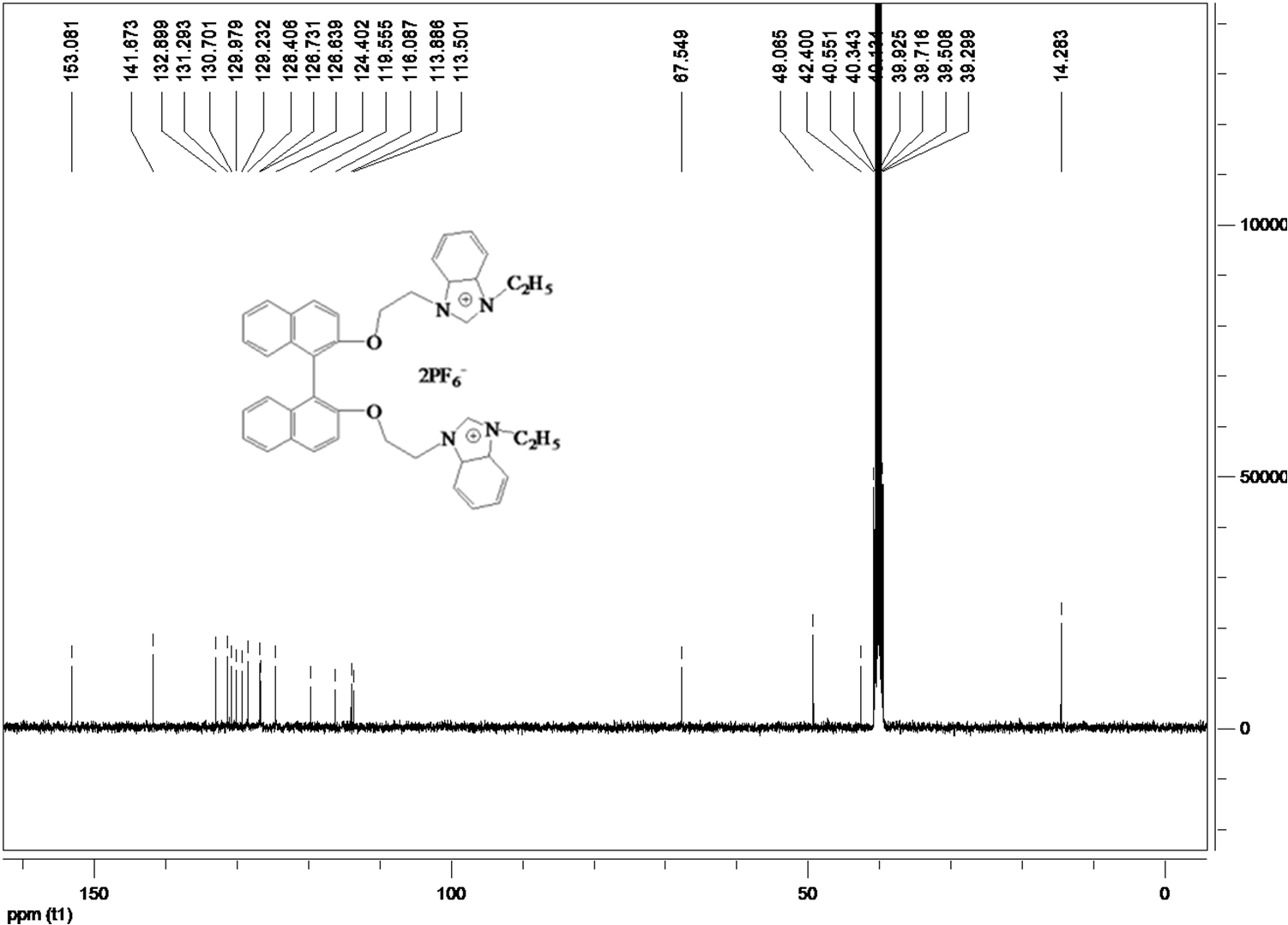
**

**Figure S21.** The ^13^C NMR (100 MHz, DMSO-*d*_6_) spectrum of **[(*S*)-L^1^H_2_]·(PF_6_)_2_**.

**
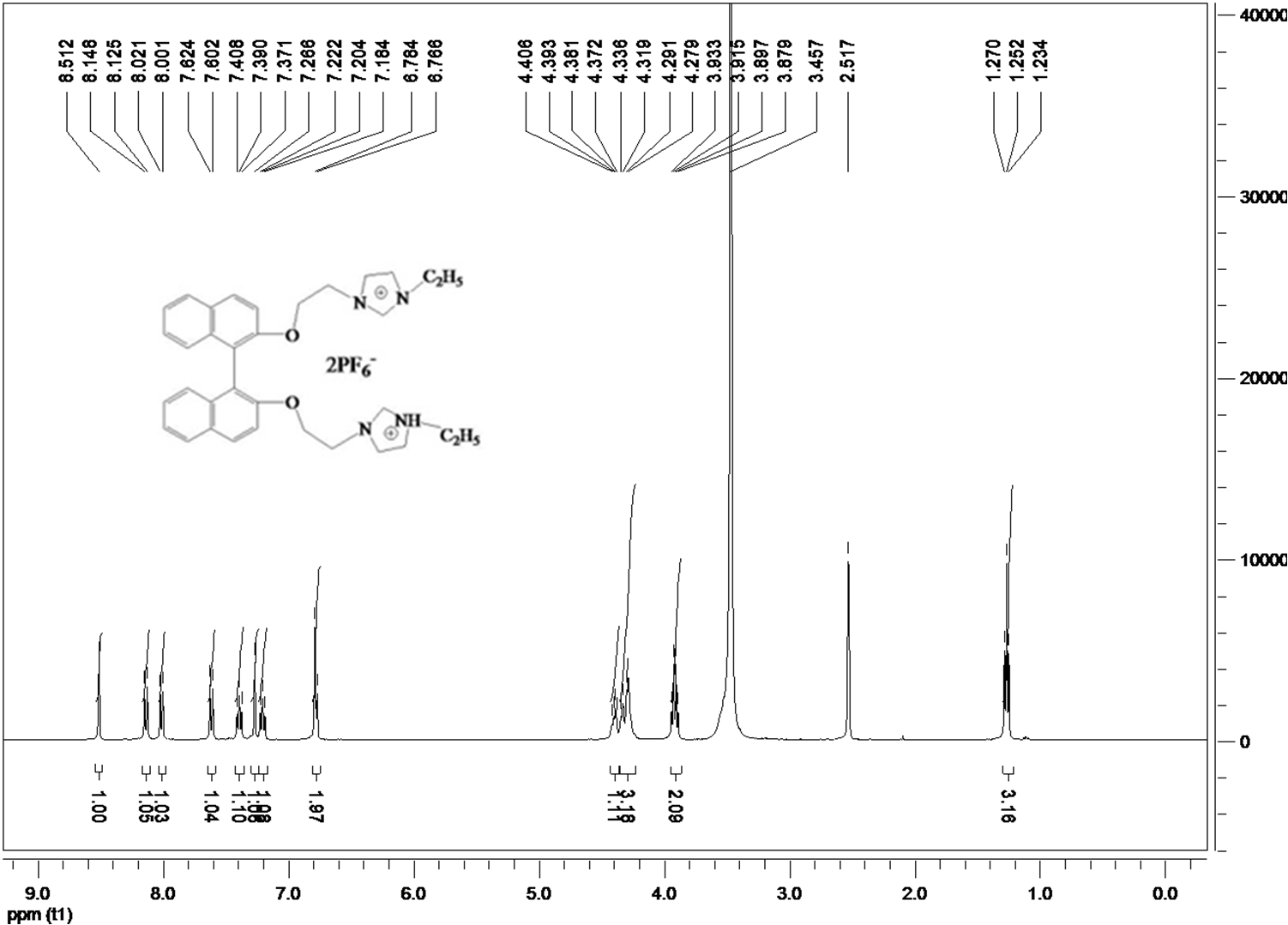
**

**Figure S22.** The ^1^H NMR (400 MHz, DMSO-*d*_6_) spectrum of **[(*S*)-L^2^H_2_]·(PF_6_)_2_**.

**
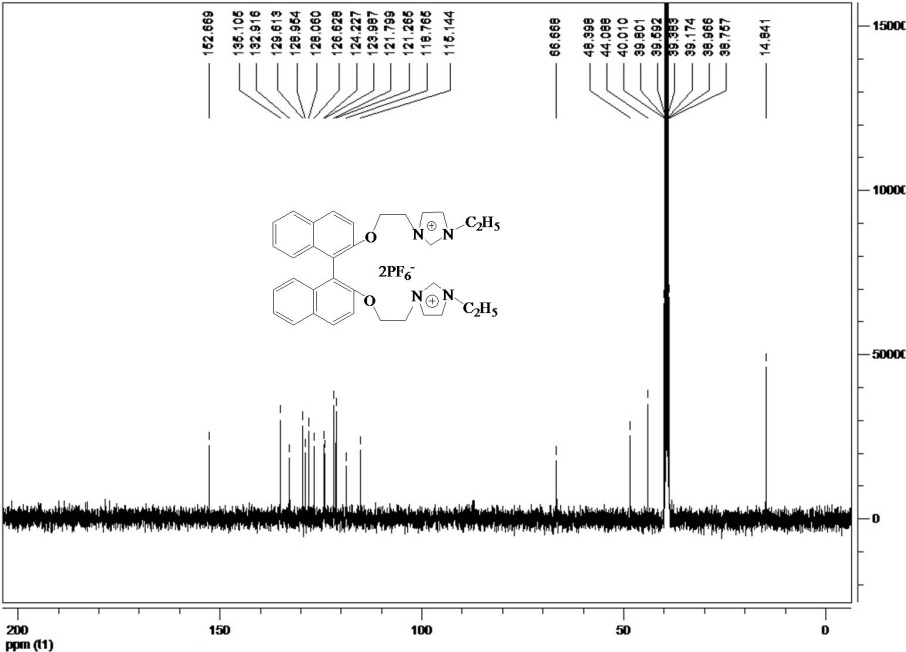
**

**Figure S23.** The ^13^C NMR (100 MHz, DMSO-*d*_6_) spectrum of **[(*S*)-L^2^H_2_]·(PF_6_)_2_**.


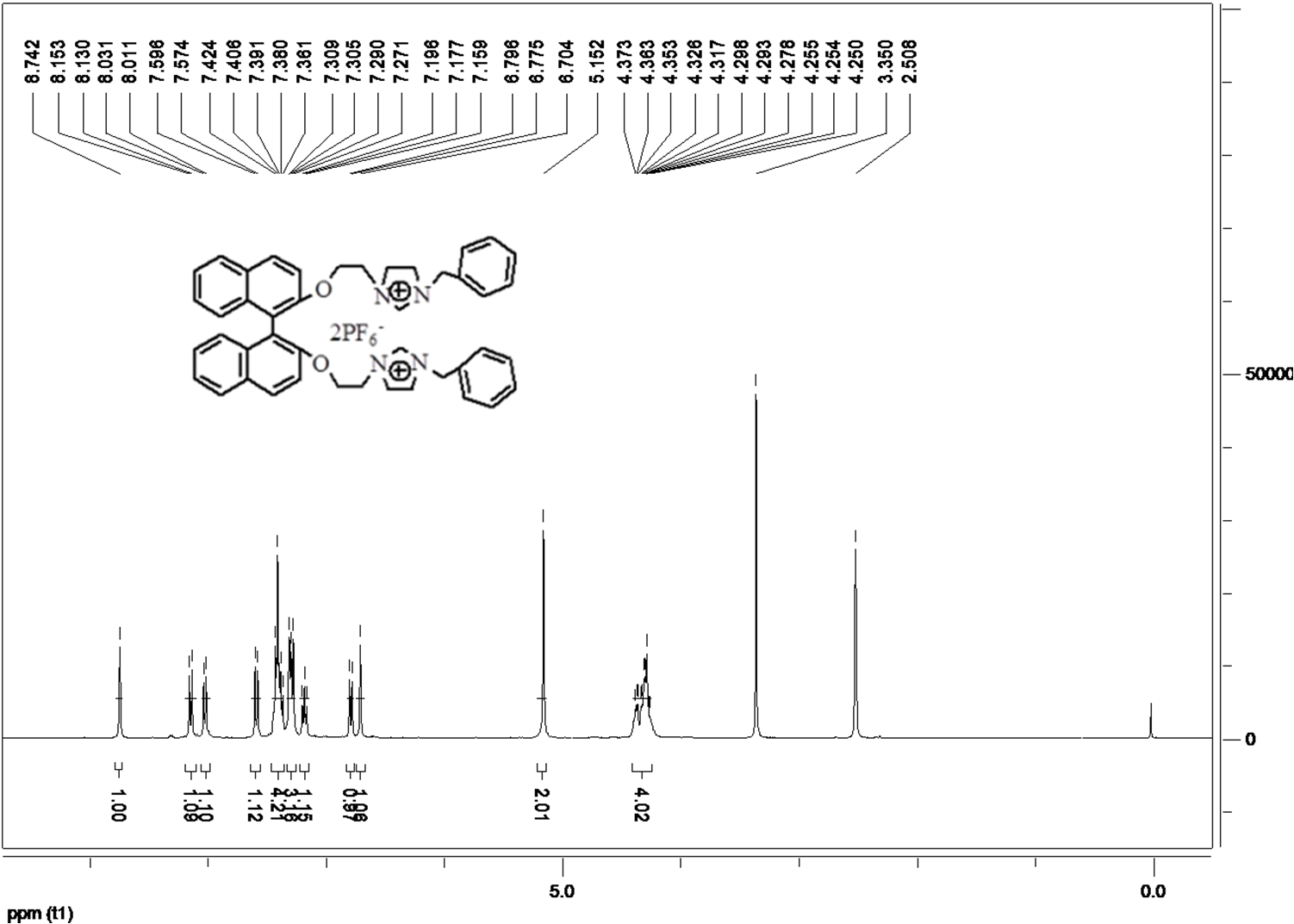


**Figure S24.** The ^1^H NMR (400 MHz, DMSO-*d*_6_) spectrum of **[(*S*)-L^3^H_2_]·(PF_6_)_2_**.


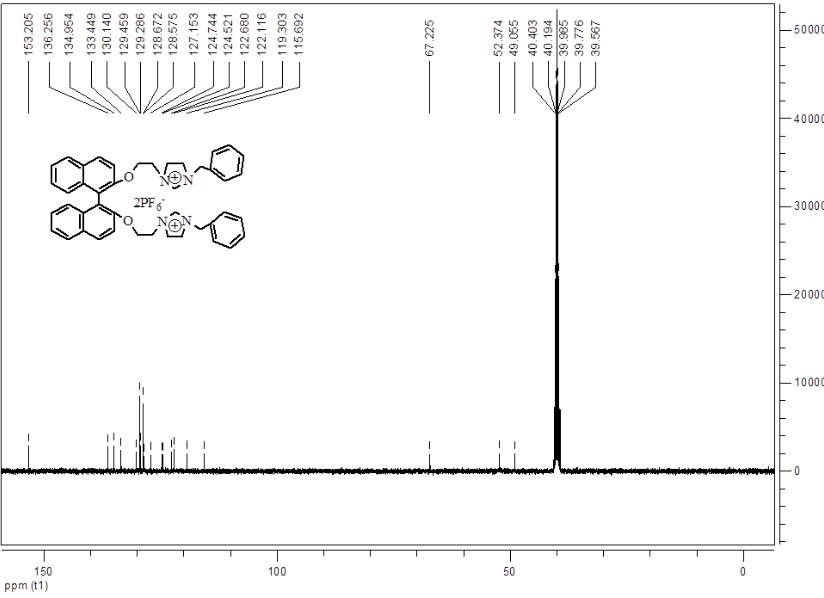


**Figure S25.** The ^13^C NMR (100 MHz, DMSO-*d*_6_) spectrum of **[(*S*)-L^3^H_2_]·(PF_6_)_2_**.

**
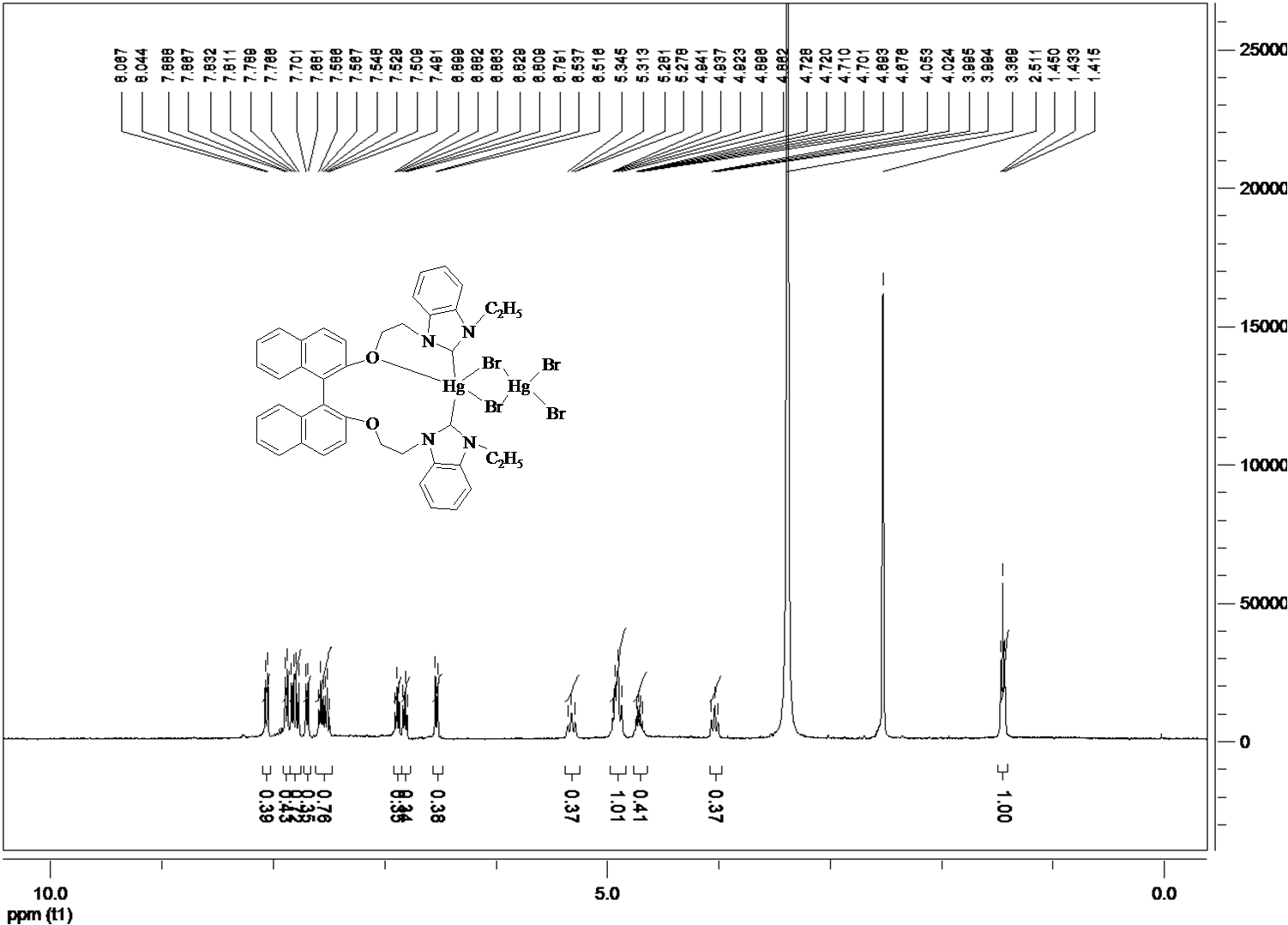
**

**Figure S26.** The ^1^H NMR (400 MHz, DMSO-*d*_6_) spectrum of **1**.

**
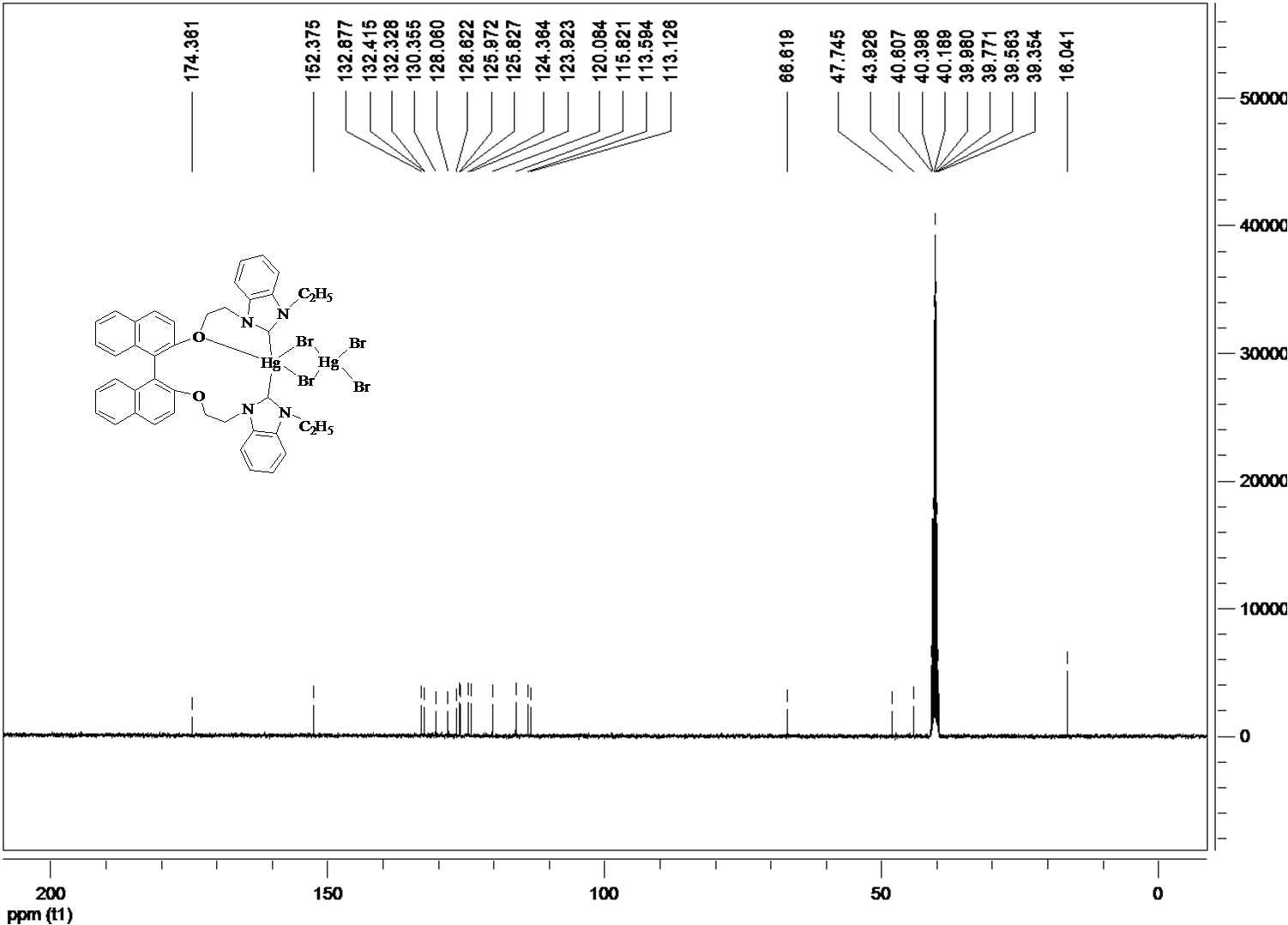
**

**Figure S27.** The ^13^C NMR (100 MHz, DMSO-*d*_6_) spectrum of **1**.

**
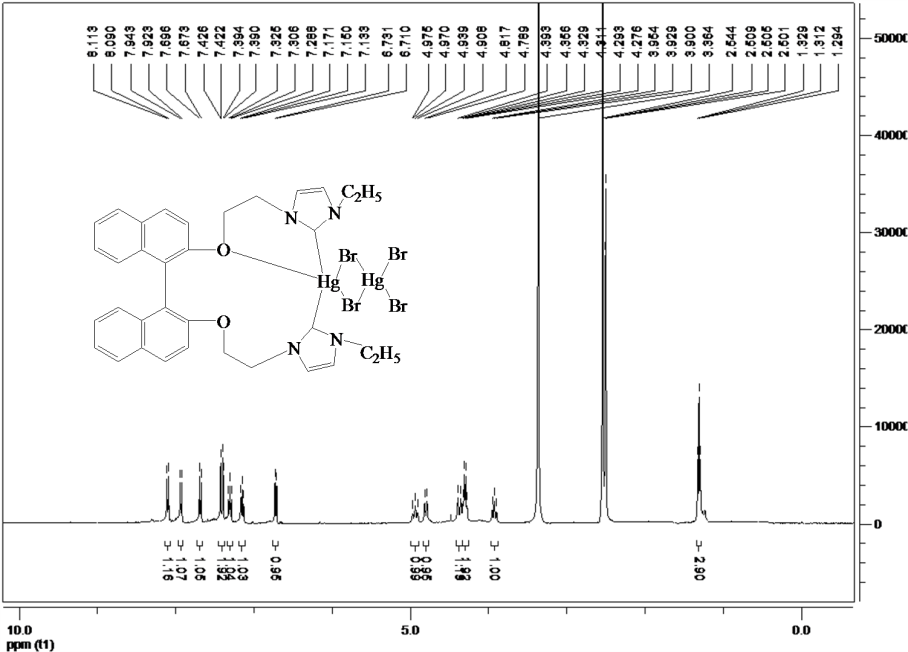
**

**Figure S28.** The ^1^H NMR (400 MHz, DMSO-*d*_6_) spectrum of **2**.

**
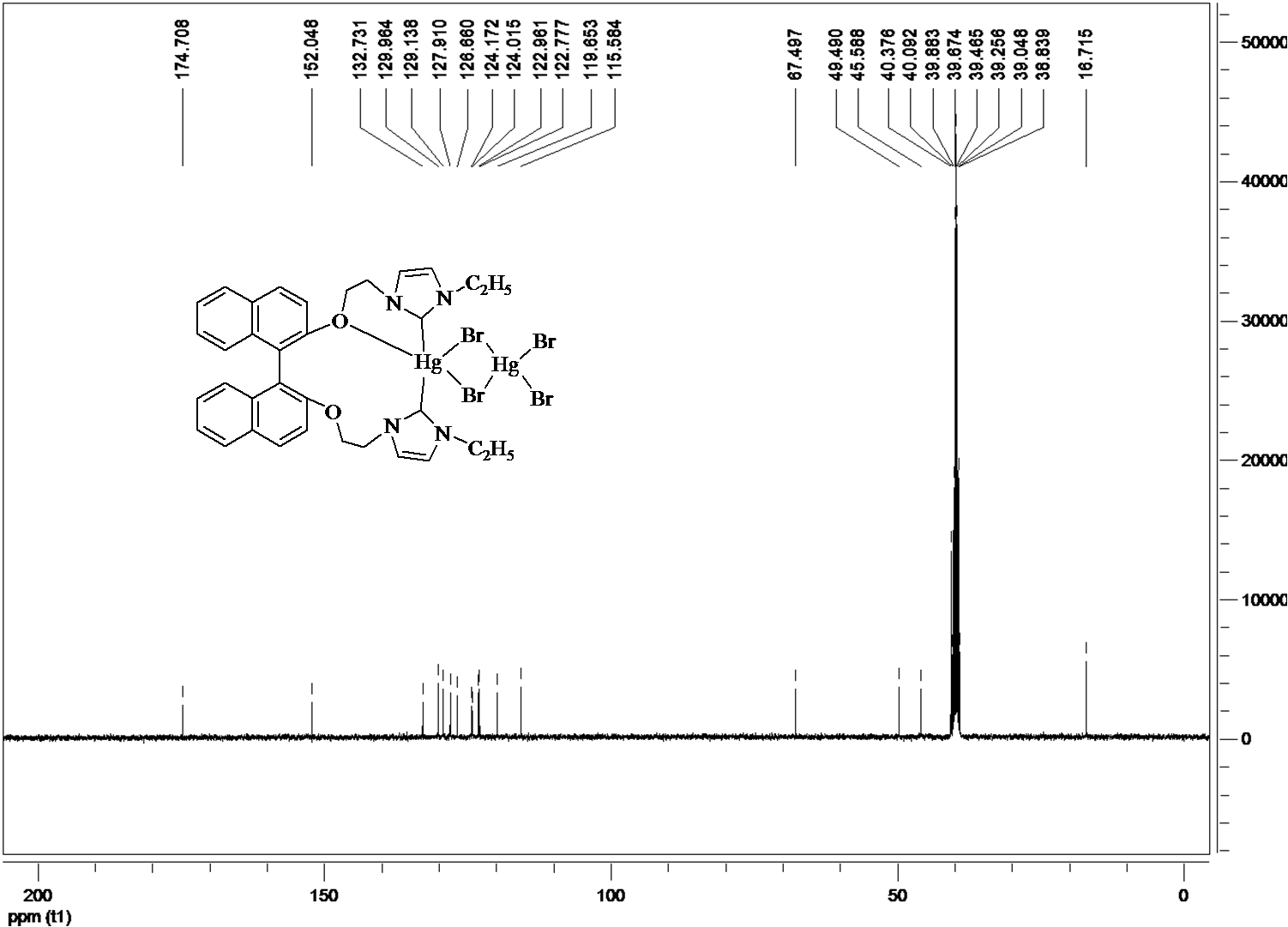
**

**Figure S29.** The ^13^C NMR (100 MHz, DMSO-*d*_6_) spectrum of **2**.

**Figure S30.** The ^1^H NMR (400 MHz, DMSO-*d*_6_) spectrum of **3**.

**
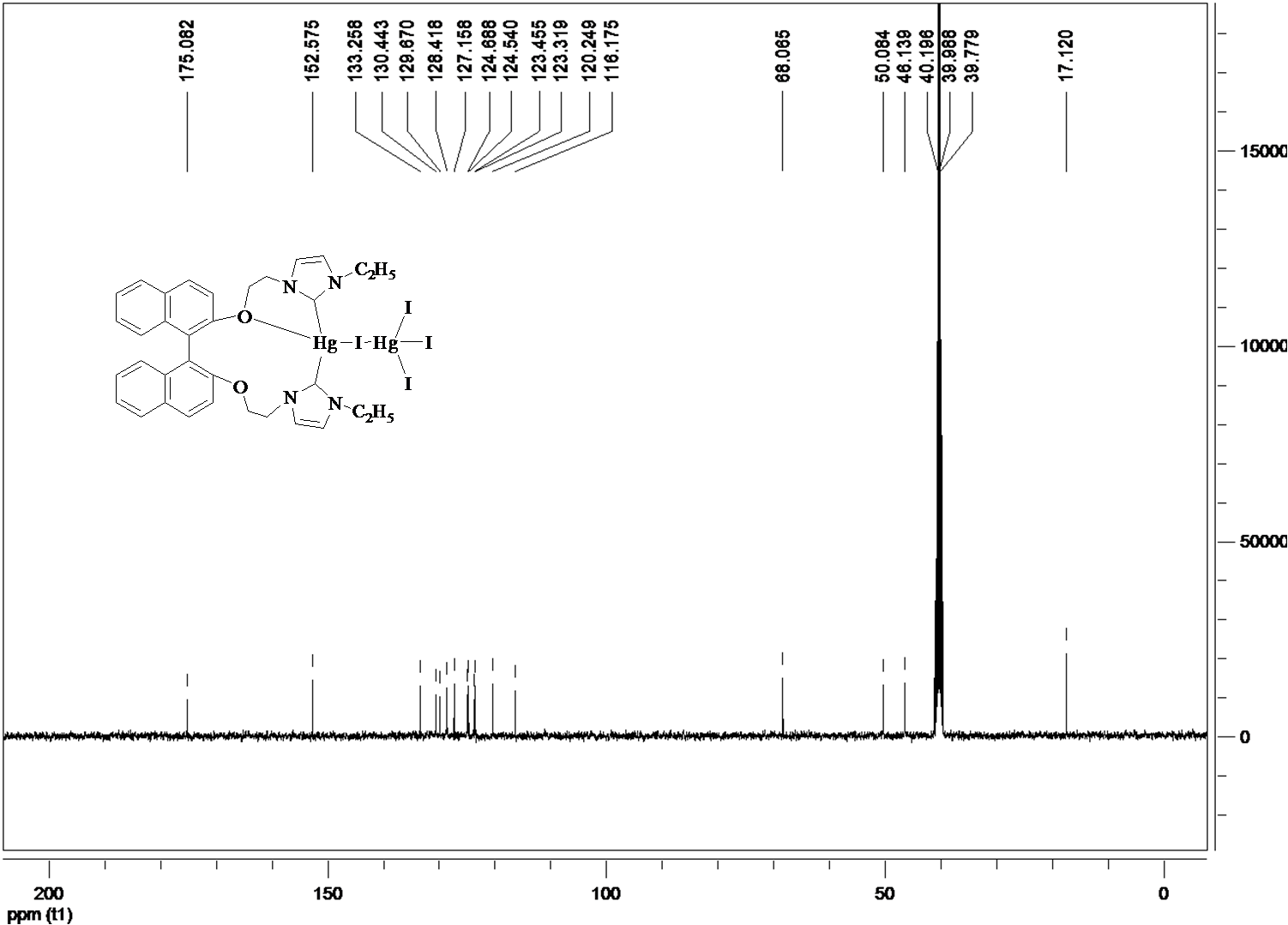
**

**Figure S31.** The ^13^C NMR (100 MHz, DMSO-*d*_6_) spectrum of **3**.

**
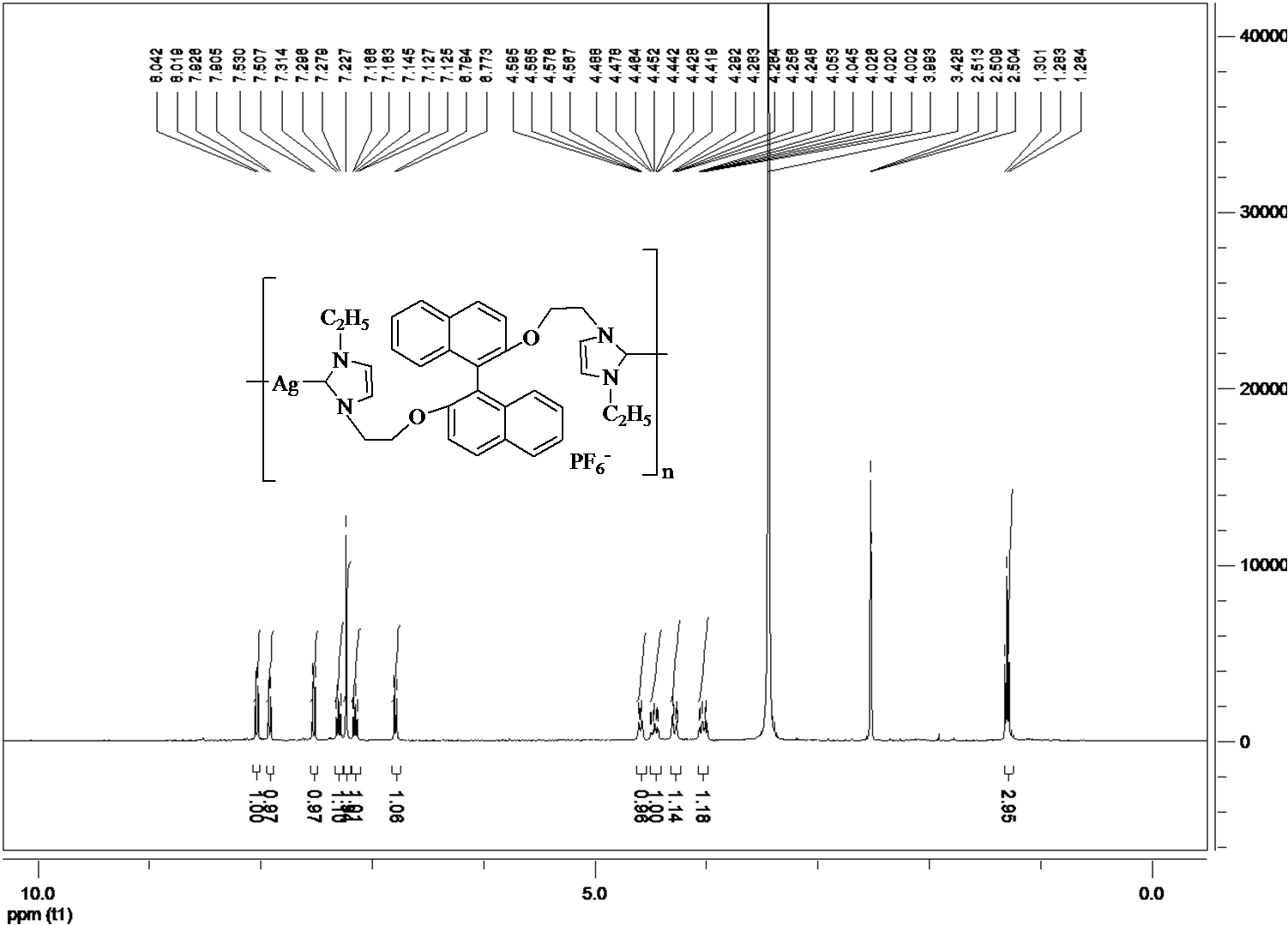
**

**Figure S32.** The ^1^H NMR (400 MHz, DMSO-*d*_6_) spectrum of **4**.

**
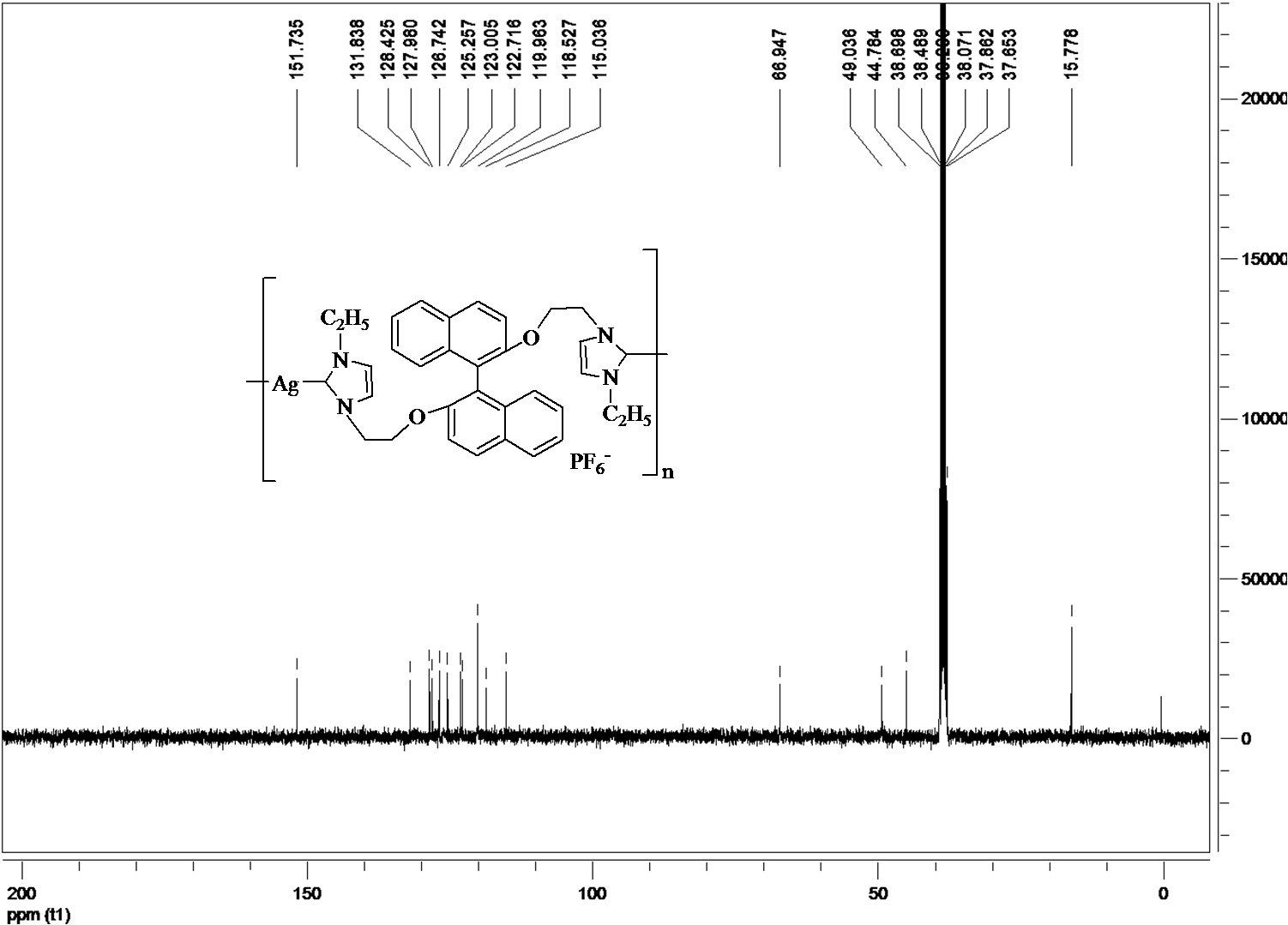
**

**Figure S33.** The ^13^C NMR (100 MHz, DMSO-*d*_6_) spectrum of **4**.


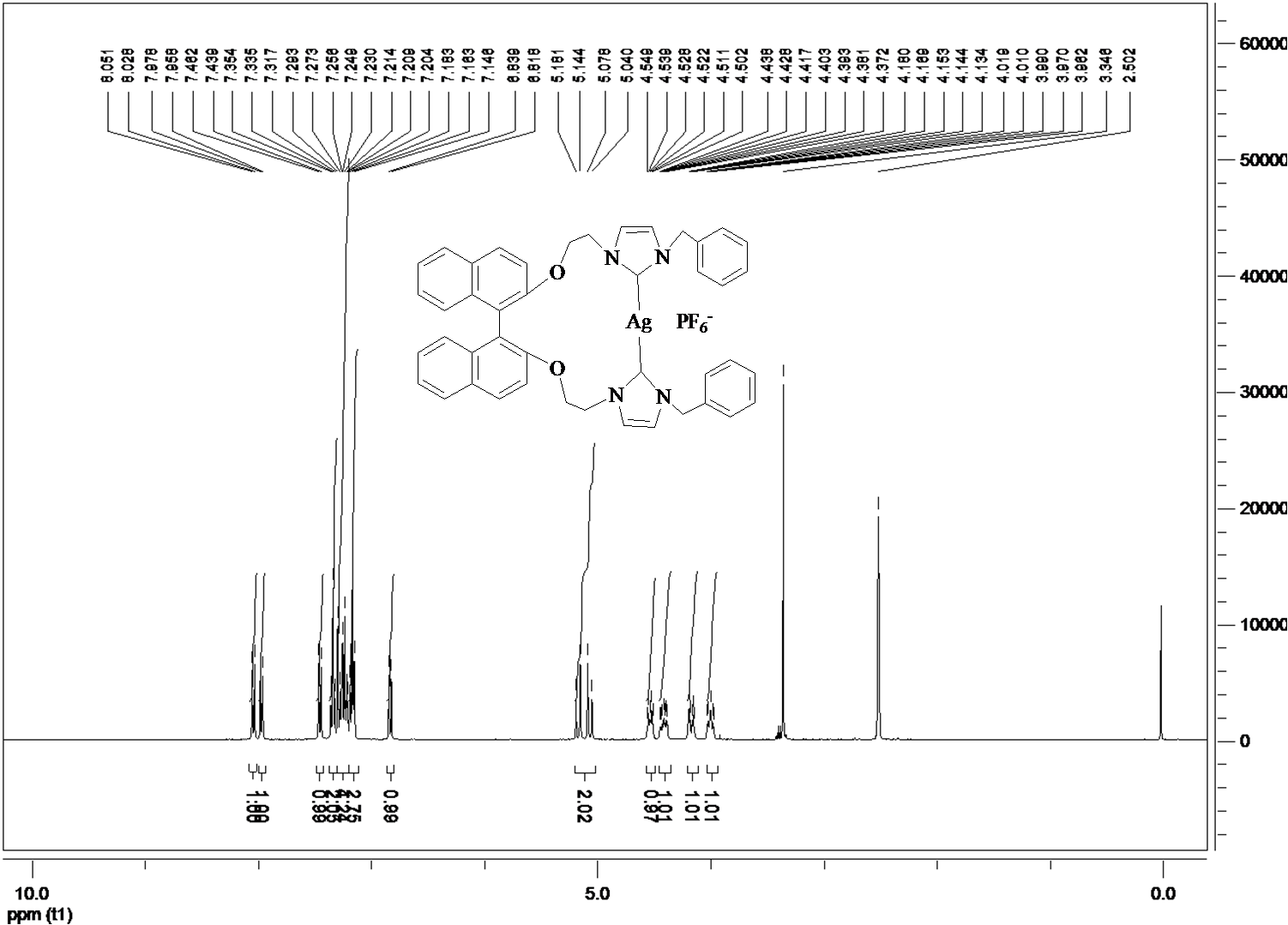


**Figure S34.** The ^1^H NMR (400 MHz, DMSO-*d*_6_) spectrum of **5**.


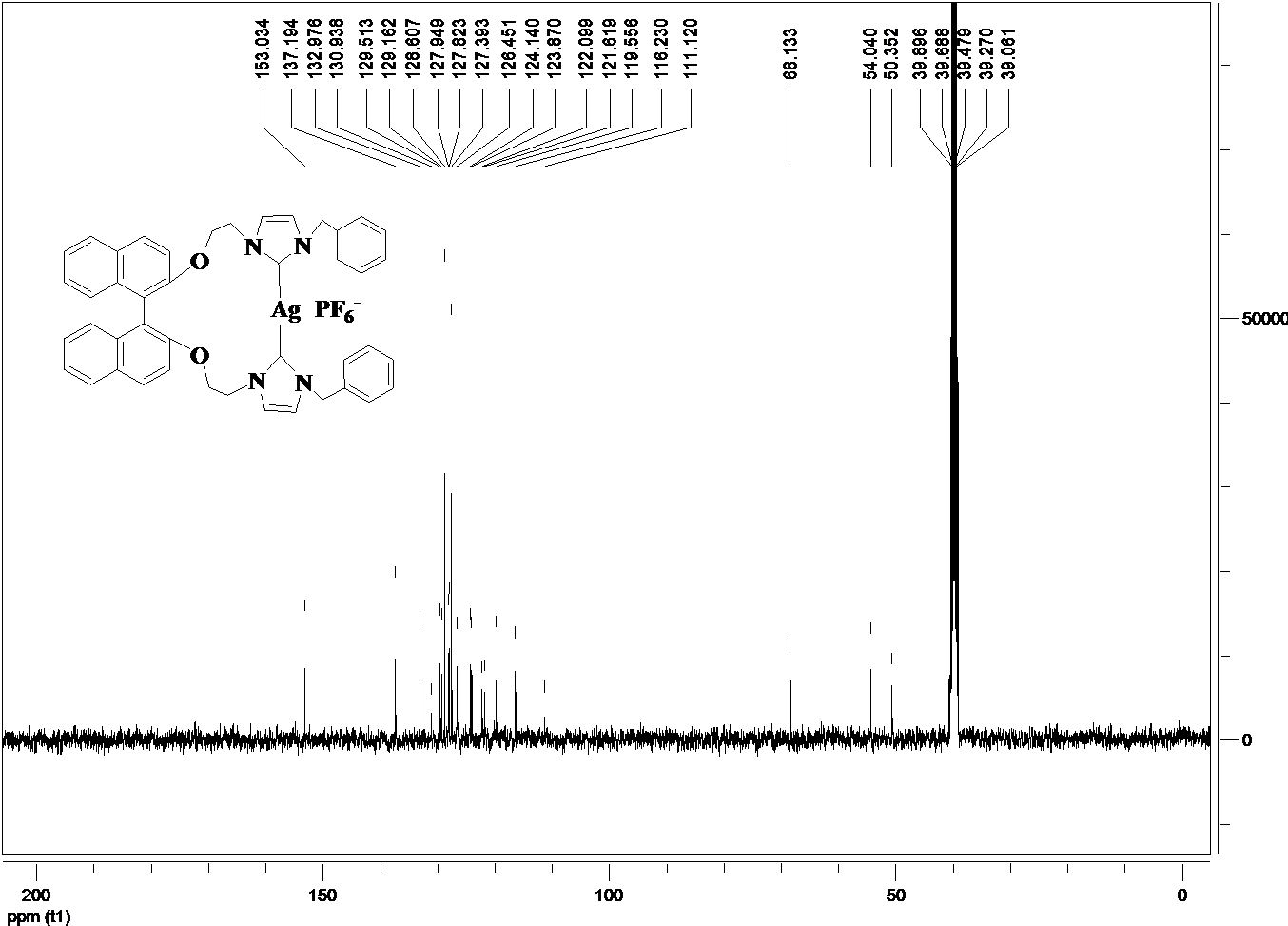


**Figure S35.** The ^13^C NMR (100 MHz, DMSO-*d*_6_) spectrum of **5**.
